# Genome-wide association study of chlamydia reinfection in African American women

**DOI:** 10.3389/fimmu.2025.1594317

**Published:** 2025-09-25

**Authors:** Hemant K. Tiwari, Amit Patki, Vinodh Srinivasasainagendra, Sandeep C. Vejandla, Archish Sadeesh, Kanupriya Gupta, Mary Appah, William M. Geisler

**Affiliations:** ^1^ Department of Biostatistics, School of Public Health, University of Alabama at Birmingham, Birmingham, AL, United States; ^2^ McMaster University, Hamilton, ON, Canada; ^3^ Division of Infectious Diseases, Department of Medicine, Heersink School of Medicine, University of Alabama at Birmingham, Birmingham, AL, United States

**Keywords:** *Chlamydia trachomatis*, GWAS, bacteria, reinfection, eQTL, chromatin interactions, cell-specific expression

## Abstract

**Background:**

*Chlamydia trachomatis (Ct)* is a bacterium that causes chlamydia, the most diagnosed bacterial sexually transmitted infection (STI) in the world. In the U.S., chlamydia is most prevalent among non-Hispanic African American (AA) individuals, implying substantial racial disparity. Despite prevention and control efforts, reinfection is common, suggesting that some individuals have insufficient protective immunity to *Ct*. To better understand the genetically mediated risks of chlamydia reinfection, we sought to identify genetic loci associated with reinfection using a Genome-Wide Association Study (GWAS) approach.

**Method:**

We performed GWAS in 300 AA women with versus without chlamydia reinfection based on *Ct* testing done about 3 months after chlamydia treatment. We conducted logistic regression models to test the additive genetic effect and used Firth regression to confirm the association results. Furthermore, we performed post-GWAS analysis to determine the functional consequences of GWAS hits, including fine-mapping, expression quantitative loci (eQTL) and chromatin interaction analyses, tissue and cell-type expression, and pathway analysis.

**Results:**

GWAS identified 17 suggestive genomic regions of interest. Five genomic regions out of 17 were identified as strongly associated with reinfection, using linkage disequilibrium and fine mapping. The positional mapping, eQTL, and chromatin interactions (CIs) analyses further identified 12 gene targets. Among the 12 gene targets, *CHIT1*, *ADORA1*, and *CHI3L1* in chromosome 1 (chr. 1); *TDRP*, *FBXO25*, and *SULF1* in chr. 8; and the *SOCS6* gene in chr. 18, were functionally relevant to reinfection.

**Conclusions:**

This GWAS study in AA women identified multiple novel genes associated with chlamydia reinfection, including *CHIT1, CHI3L1, ADORA1, ALK, TDRP, FBXO25, LINC01592, SULF1*, and *SOCS6*, which are involved in the immune response. *CHIT1, ADORA1, CHI3L1, TDRP, FBOXO25*, *SULF1*, and *SOCS6* were identified using CI/eQTL mapping.

## Introduction

Chlamydia, the most diagnosed bacterial sexually transmitted infection (STI), is caused by the bacterium *Chlamydia trachomatis* (*Ct*). In 2020, the World Health Organization estimated there were 128.5 million new chlamydia infections among adults (15–49 years old) globally (1). According to CDC’s *Sexually Transmitted Infections Surveillance, 2022*, there were ~1.7 million new cases in the U.S (2).; furthermore, chlamydia affects African American (AA) females disproportionately, as they have almost a 5-fold higher chlamydia infection rate than Caucasians (3). Chlamydia infections are more prevalent in women than men (1). Chlamydia in women is of particular concern because ascending infection into the reproductive tract can cause pelvic inflammatory disease (PID), which may lead to scarring in the fallopian tubes that can result in infertility and chronic pelvic pain as well as an increased risk for ectopic pregnancy (4–9). Chlamydia is also associated with pregnancy complications (e.g., preterm labor and stillbirth), neonatal infection (conjunctivitis and pneumonia), and an increased HIV transmission risk (10–12).

Despite prevention and control efforts, reported chlamydia cases in the US remain high, and chlamydia reinfection within a year after treatment is common (up to 20%), suggesting that some infected persons do not develop sufficient immune protection to *Ct* and/or it is short-lived (4, 13–19). In a study evaluating British Columbia chlamydia surveillance data, reinfections accounted for a significant proportion of reported annual cases, and the rise in annual reinfection rates paralleled the rise in total chlamydia cases, stressing the important contribution of reinfections to rising chlamydia rates (20). Thus, there is an urgent need to understand why some are prone to chlamydia reinfection compared to others. This includes identifying specific immune responses and genetic variants that may act as protective or risk factors for reinfection.

Animal models, mostly murine, have shown that CD4^+^ IFN-γ response against chlamydia (i.e., CD4^+^ T-cells that secrete IFN-γ in response to chlamydia) is essential for chlamydia clearance and provides protection against reinfection (21–25). Some human studies have validated that *Ct*-specific CD4^+^ IFN-γ is a correlate of immunity to reinfection (26–28). Bakshi et al. showed that most IFN-γ-producing CD4^+^ T cells in women without reinfection were polyfunctional, usually co-producing TNF-α, suggesting that TNF-α may also be an important cytokine in immunity to *Ct* (26). CD4^+^ effector responses can be influenced by HLA class II molecules on antigen-presenting cells. Associations of HLA class II alleles with *Ct* infection outcomes have been reported (29–37), but are inconsistent, except for DQB1*06, which has been reported as a risk marker for *Ct* incidence, reinfection, PID (29–31, 35, 36), and infertility (34). In a genetic study from the REACH multicenter cohort, Wang et al. reported a 2.1-fold higher chlamydia reinfection risk in those with DQB1*06 (35), and this association was then confirmed in another cohort enrolled in Birmingham, AL (OR 2.7) (31). We recently performed next-generation sequencing to map HLA class II variants spanning the *HLA-DQ* and -*DR* loci in this same Birmingham, AL, cohort and found that DQB1*06 and DQB1*04 were significant predictors of *Ct* reinfection, and *DRB1*, *DRB5*, *DQA2*, and three intergenic regions also had variants associated with reinfection (29).

However, there is a dearth of knowledge regarding the role of non-HLA genes in chlamydia reinfection. Genome-wide association studies (GWAS) in human chlamydia infections are sparse. In a GWAS study by Roberts et al. in Dutch women who were *Ct* seropositive (cases) *vs*. high-risk *Ct* seronegative (controls), they identified two candidate gene regions associated with *Ct* seropositivity: cyclic GMP-dependent protein kinase (*PRKG1*) gene and the G protein-coupled receptor (*NPSR1*) gene (38). Their findings imply that these signaling pathways may influence the innate immune response to *Ct* exposure and risk for infection acquisition. In 2021, Zheng et al. performed a GWAS on *Ct*-related infertility in women and identified 112 candidate infertility loci and 31 related to *Ct* ascension (39). The single nucleotide polymorphisms (SNPs) identified in the study were found to influence chlamydial ascension by modulating the expression of 40 mediator innate immunity genes, including type I interferon production, T-cell function, fibrosis, female reproductive tract health, and protein synthesis and degradation (39). Furthermore, in 2022, Zhong et al. identified genetic loci susceptibility to *Ct* upper genital tract infection in women (40). They identified cis-eQTLs that modulate mRNA expression in 81 genes correlated with an altered risk of ascending infection. Genes involved in proinflammatory signaling were upregulated, while genes related to T cell functions—crucial for chlamydial control—were downregulated, in women with endometrial infection (40).

To better understand the genetically mediated risk of chlamydia reinfection, we performed a GWAS study to shortlist putative SNPs and genes associated with reinfection using linkage disequilibrium and investigated the extent of significance around the significant SNPs by fine mapping to identify possible causal SNPs associated with reinfection. We also investigated the involvement of candidate SNPs in expression quantitative loci (eQTL) and chromatin interactions (CIs) in *silico* to study the biologically functional aspect of the candidate loci. Lastly, we investigated the joint effect of GWAS gene expressions in tissues, single-cell types, gene set analysis, and pathway analysis.

## Materials and methods

### Study sample

Genomic DNA was previously collected from women who presented to a sexual health clinic in Birmingham, AL, for treatment for a positive screening *Ct* nucleic acid amplification test (NAAT) and were enrolled in a chlamydia immunogenetics study as described in detail elsewhere (31, 41). Briefly, at enrollment, the women provided written consent, were interviewed, had blood and urogenital specimens collected, and received directly observed chlamydia treatment (azithromycin). They then returned for 3-month and 6-month follow-up visits, during which time the interview and collection of specimens were repeated, and reinfection was assessed by *Ct* NAAT. The study was approved by the University of Alabama at Birmingham Institutional Review Board and the Jefferson County Department of Health. Most study participants reported as African American (AA) race. Our GWAS study focused on the 300 AA women with reinfection data available and sufficient DNA for GWAS.

### Genotype and quality control

Stored genomic DNA was genotyped on Illumina Global Diversity Array v1.0 with 1,882,945 variants. We evaluated and removed the variants that had more than 5% missing data, less than 5% minor allele frequency, or a Hardy-Weinberg Equilibrium (HWE) test P-value less than 1×10^-5^, leaving 748,059 variants for further analysis. Although participants were expected to be unrelated, we assessed their relatedness by calculating identity by state (IBS) using the KING software (version 2.2.7) (42). We also confirmed self-reported gender with genetically estimated gender using PLINK software version 2.0 (43). We calculated principal components (PCs) using the EIGENSOFT program (44), by selecting only tag variants (55,061) based on pairwise linkage disequilibrium (LD) less than 0.05. We investigated population admixture with 1000G populations using Principal Component Analysis (**Supplementary Figure S1**). The top 10 principal components were used to identify and remove genetic outliers using the Mahalanobis distance method (45, 46). Along with the final GWAS dataset, analyses also included clinical covariates such as age, unprotected sex since the last visit, and new partner since the last visit. Furthermore, we estimated African and European ancestry for the samples to include as a covariate in the model using ADMIXTURE software version 1.3.0 (47, 48).

### GWAS imputation

McCarthy Group Tools were used for pre-imputation quality control (49). Using TOPMed as a reference panel, the alleles were corrected for strand consistency. After strand correction, SNPs were removed if the allele frequency for A/T & G/C SNPs differed by more than 0.4, and for other SNPs by more than 0.2. We imputed genotypes on the TOPMed imputation server (https://imputation.biodatacatalyst.nhlbi.nih.gov/#)! using NHLBI TOPMed (release 2, cosmopolitan samples) as the reference panel. We eliminated poor quality variants based on the imputation quality metric R^2^ < 0.8 and expected allele frequency (EAF < 0.05). Subsequently, the post-imputation variants were re-evaluated for missingness, EAF, and HWE. After removing variants with more than 5% missingness, or less than 5% EAF, or HWE P-value < 1×10^-5^, 8,701,728 variants remained for association analysis.

### Association analysis

We performed logistic regression models with chlamydia reinfection status as an outcome and variants as predictors with age, ancestry, a new sex partner since the last visit, and unprotected sex since the last visit as covariates using PLINK 2.0 to detect SNPs associated with *Ct* reinfection, adjusting for potential confounding effects (43). We used a quantile-quantile (QQ) plot to investigate the genomic variance inflation in the GWAS. The logistic regression results were then confirmed with Firth regression using PLINK. The Firth logistic regression provides bias reduction for small sample sizes and yields finite and consistent estimates (50, 51). Firth’s logistic regression uses a penalized likelihood approach to reduce bias from the maximum likelihood estimates in the logistic regression model, resulting in well-calibrated Type 1 error.

### Annotation

The SNPs from the GWAS were annotated using ANNOVAR to determine both gene and SNP-level functions (52, 53). The dbSNP151 data release from UCSC was employed to assign rs# IDs to our variants reported in the supplementary results dataset. To address discrepancies in SNP locations between the human genome builds hg19 and hg38, functional annotations for both hg19 and hg38 are cataloged in all **Supplementary Tables**.

### Post GWAS analyses

Post-GWAS (Post-Genome-Wide Association Studies) research is crucial because while GWAS identifies statistical associations between genetic variants and phenotype, it doesn’t explain how or why those variants affect the phenotype. Post-GWAS studies aim to translate these associations into biological understanding. Post-GWAS helps to identify causal variants, regulatory elements, and their target genes by eQTL mapping and Chromatin interaction mapping to find target genes since most associated SNPs are in non-coding regions (e.g., enhancers, promoters). Also, including GWAS genes and targeted genes in pathway and network analyses enables the identification of biological pathways or networks affected by genetic variation that provide insight into potential disease mechanisms.

### LocusZoom plots

We used LocusZoom plots to display regional genomic information relative to significant index SNPs, including the statistical association strength and extent of the association signals of nearby SNPs, local linkage disequilibrium (LD) and recombination patterns, and the positions of genes in the region (54, 55). We used the LD patterns in and around +/- 200kb from the base pair location of the significant variant. All pair-wise LD for the variants in the +/- 200kb region was calculated using PLINK2.0 for all variants with P <0.05 (43).

### Fine-mapping

Note that not all associated SNPs are causal, but a GWAS SNP may be in LD with the true causal variant. Fine mapping helps in detecting true causal variant(s) that functionally influence the phenotype. In our fine-mapping analysis, we used the PAINTOR v.3.0 software package to discover potential causal variants by leveraging the GWAS summary statistics, LD, and well-curated functional hot-spot regions of the genome (56–58). After carefully studying the LD patterns around lead significant variants, we followed up with fine mapping for significant variants after LocusZoom. Like LocusZoom, variants from our GWAS were centered within +/- 200kb of the lead variants with GWAS P <0.05. We used the approach showcased in the PAINTORv3 fine-mapping software distributed through the GitHub repository to determine tissue-based annotations for fine mapping. Although PAINTOR is enriched for 8000+ annotation tracks representing different combinations of tissues and genomic regions, our primary focus was on annotation tracks related to these keywords: Ovary, Ovaries, Uterus, Uterine, CD4, CD8, Cervix, Cervical, Rectal, Placenta, Breast, Vagina, Colon, T-Cell and B-Cell. This yielded a total of 1,448 annotation tracks. The sum of the log-Bayes factors (BFs) and effect size estimates for each annotation is converted into the relative probability of an SNP being causal in a given annotation track. To assess annotation significance, the sum of the BFs for the baseline annotation was compared with both the baseline and each of the selected annotations. The statistical significance of the enrichment was then calculated using a ratio test. The likelihood ratio test (LRT) was used to evaluate each annotation. We selected the top 10 annotations to calculate the posterior probability of each SNP, which contains the top GWAS SNPs.

### Functional mapping and annotation of genome-wide association studies

The goal of the FUMA analysis is to decipher the biological and regulatory potential of the GWAS SNPs. FUMA provides annotations of SNPs with their biological functionality and maps them to genes based on physical distance (10kb window) from known protein-coding genes, known eQTL, i.e., significant association between SNP and differentially expressed genes for quantitative traits, and existing chromatin interaction (CI) information. CI mapping can involve distal chromatin markers from the sentinel SNP, and the interaction region can span multiple genes. Specifically, the SNP2GENE module provides eQTL information, CI, a heatmap of gene expression, tissue specificity (DEG), overrepresentation in gene sets, and cell type specificity of the significant genes for chlamydia reinfection GWAS. We conducted functional and biological relevance analyses of significant coding and non-coding SNPs using the SNP2GENE and GENE2FUNC modules of FUMA GWAS (59, 60). FUMA integrates several biological data repositories and tools to process input GWAS summary statistics. SNPs are annotated with their biological functions and mapped to genes based on positional data, eQTLs, and chromatin interaction information in the SNP2GENE module of FUMA. The SNP2GENE module provides a heatmap of gene expression, tissue specificity, differentially expressed genes (DEGs), and overrepresentation in gene sets. In addition, we used FUMA to investigate the cell type specificity of the significant genes for chlamydia reinfection GWAS.

### Gene and pathway-based analysis

Pathway analysis is important because it connects genetic associations to biological mechanisms by examining groups of functionally related genes, providing deeper and more interpretable insights than analyzing individual variants or genes alone. We performed gene-based association and VEGAS2Pathway analyses using VEGAS2 software (61, 62). VEGAS2 is an extension of the VErsatile Gene-based Association Study (VEGAS) approach, which uses 1000 Genome populations to estimate patterns of linkage disequilibrium for each gene.

### GWAS SNPs single cell expression in mouse and human cells

Studying GWAS SNPs in single-cell expression data from mouse and human cells is crucial because it helps reveal how genetic variants influence gene regulation at the cellular level. This is essential for understanding the mechanistic basis of phenotype to identify the most relevant SNPs for follow-up. We used FUMA cell type specificity analyses with single-cell RNA-seq (scRNA-seq) (59, 60). FUMA uses MAGMA gene-property analysis with scRNA-seq data (63). We used Mouse Cell Atlas in FUMA to implicate cell type specificity with chlamydia reinfection GWAS SNPs. In addition, we used the Phenotype-Cell-Gene-Association Analysis (PCGA) platform to investigate cell-type expression corresponding to chlamydia reinfection GWAS SNPs in human cell types (64–66). PCGA is a web server that simultaneously estimates associated tissues/cell types and genes of complex diseases and traits using GWAS summary statistics. PCGA contains 54 human tissues, 2,214 human single-cell types, and 4,384 mouse single-cell types.

## Results

### Characteristics of the study sample

Of the 300 AA women evaluated for inclusion in our GWAS study, we removed 11 women for being either first-degree relatives, having gender misclassification, or being genetic outliers. In addition, 6 were removed since they did not have data on either a new partner since the last visit or unprotected sex since the last visit. After quality control, we retained 283 AA women, 57 with reinfection and 226 without reinfection, with complete data for outcome and covariates age, unprotected sex since the last visit, and new partner since the last visit. The covariate characteristics stratified by reinfection status are shown in **Supplementary Table S1A**. The mean (± SD) age of the subjects with reinfection was 22.65 (± 3.56) years, while the mean age of those without reinfection was 23.99 (± 4.90) years (P = 0.021). Furthermore, we performed association analysis of *Ct* reinfection with clinical covariates, using logistic regression (**Supplementary Table S1B**). Note that none of the covariates were statistically significant. We estimated African ancestry proportions to include as a covariate in the model to correct for any admixture. Ancestry estimates of AA women are depicted in **Supplementary Figure S2**. The average (± SD) African ancestry in the sample was 0.76 (± 0.17) with a minimum of 0.15 and a maximum of 1.00.

### GWAS of chlamydia reinfection

**Figure 1:**
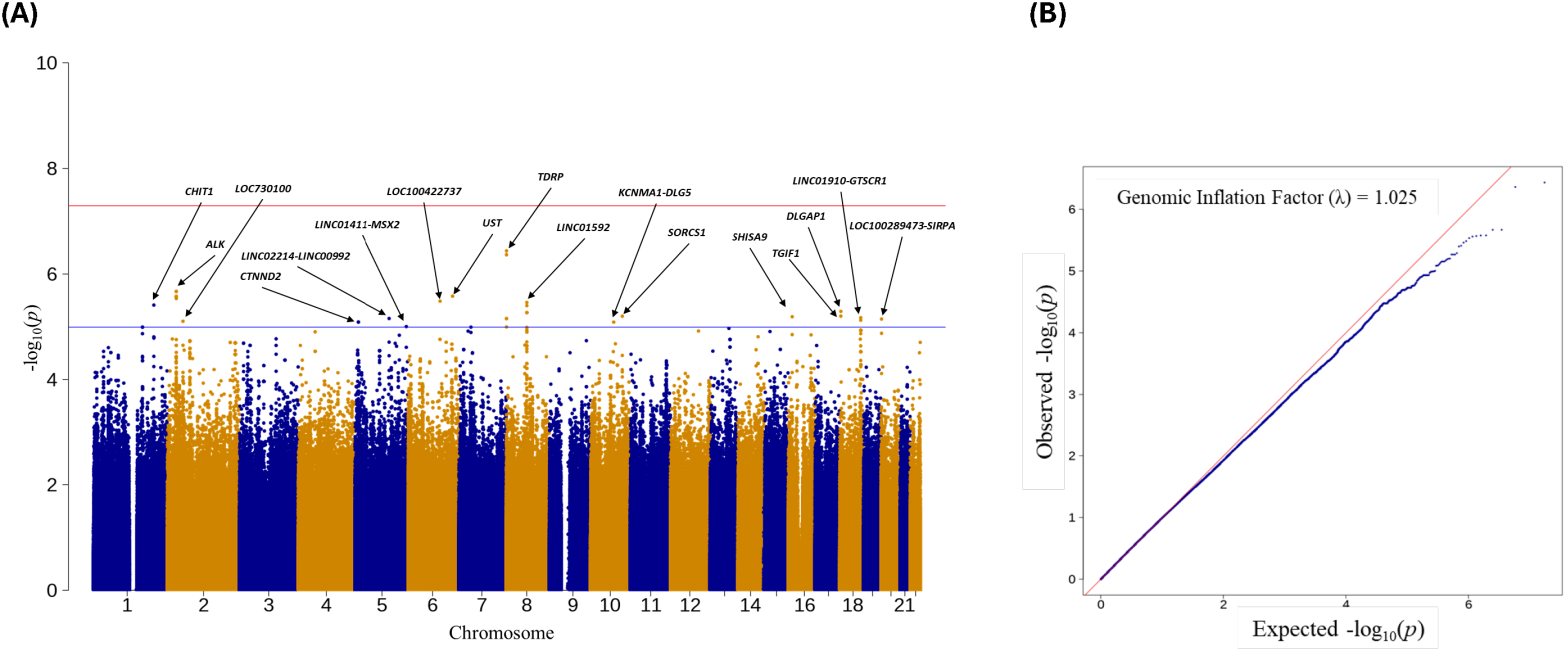
**(A)** Manhattan plot of Ct reinfection GWAS. Names of the 17 genes/genomic regions with P-value<1.0E-05; **(B)** QQ-plot of Ct GWAS results with a genomic inflation factor.

A Manhattan plot provides a visualization of the -log(P-values) distribution across the entire genome, and a quantile-quantile (QQ) plot is used to assess whether the observed P-values distribution aligns with the expected distribution under the null hypotheses of no association. Manhattan and QQ plots for chlamydia reinfection GWAS are depicted in **Figure 1**, adjusted for age, ancestry, new sex partner, and unprotected sex since the last visit. We found several significantly suggestive SNPs within or close to the gene (See **Figure 1**). In particular, SNPs in *CHIT1* (chromosome 1); *ALK* and *LOC730100* (chromosome 2); *CTNND2*, *LINC00992* and *MSX2* (chromosome 5), *LOC100422737* and *UST* (chromosome 6); *TDRP* and *LINC01592* (chromosome 8); *DLG5* and *SORCS1* (chromosome 10), *SHISA9* (chromosome 16); *LINC01910*, *DLGAP1*, and *TGIF1* (chromosome 18), and *SIRPA* and *LOC100289473* (chromosome 20) showed suggestive significance with P <1.0E-05. **Table 1** consists of logistic and Firth regression odds ratios and P-values, and only SNPs were included if they had a P-value of <1.0E-05 in both logistic and Firth regression. Note that the Firth regression P-values were close to those of the logistic regression. **Supplementary Table S2** contains detailed information on GWAS results with P <1.0E-05 from logistic regression and corresponding Firth regression P-values, including build 37 and build 38 coordinates, HWE P-values, and MAF comparison with 1000G populations. **Supplementary Table S3** contains the association results for all SNPs with logistic regression P<1.0E-02.

**Table 1:** SNPs associations with *Ct* reinfection outcome GWAS*.

Chr	BP Build 38/37	rsID	Function	Gene	Effective Allele	Ref. Allele	MAF	Logistic OR (95%CI)	Logistic P-value	Firth OR (95%CI)	Firth P-value
1	203,222,776/ 203,191,904	rs2486961	intronic	*CHIT1*	T	C	0.23	3.20(1.95, 5.23)	3.87E-06	3.09(1.90, 5.03)	5.53E-06
2	29,718,920/ 29,941,786	rs77791547	intronic	*ALK*	G	C	0.26	3.17(1.96, 5.14)	2.69E-06	3.06(1.90, 4.93)	3.99E-06
2	29,726,905/ 29,949,771	rs17008540	intronic	*ALK*	C	T	0.26	3.24(1.98, 5.29)	2.64E-06	3.12(1.93, 5.06)	3.92E-06
2	29,727,845/29,950,713	rs111891071	intronic	*ALK*	CAG	C	0.26	3.27(2.00, 5.34)	2.13E-06	3.15(1.95, 5.11)	3.19E-06
2	29,728,121/ 29,950,997	rs66953037	intronic	*ALK*	T	TA	0.26	3.27(2.00, 5.34)	2.13E-06	3.15(1.95, 5.11)	3.19E-06
2	29,729,627/ 29,952,493	rs113164730	intronic	*ALK*	C	T	0.26	3.18(1.96, 5.16)	2.75E-06	3.07(1.90, 4.95)	4.08E-06
2	29,730,914/ 29,953,780	rs10208306	intronic	*ALK*	G	A	0.26	3.23(1.98, 5.28)	2.91E-06	3.11(1.92, 5.06)	4.32E-06
6	106,780,666/ 107,228,541	rs113237398	ncRNA intronic	*LOC100422737*	T	C	0.07	5.91(2.80, 12.50)	3.26E-06	5.63(2.68, 11.84)	5.10E-06
6	148,878,017/ 149,199,153	rs28530774	intronic	*UST*	A	G	0.06	6.51(2.98, 14.22)	2.63E-06	6.19(2.84, 13.46)	4.36E-06
8	540,864/ 490,864 490,864	rs11996757	intronic	*TDRP*	G	A	0.28	3.08(1.89, 5.04)	6.98E-06	2.98(1.84, 4.84)	9.78E-06
8	541,098/ 491,098	rs7829447	intronic	*TDRP*	C	T	0.28	3.08(1.89, 5.04)	6.98E-06	2.98(1.84, 4.84)	9.78E-06
8	546,505/ 496,505	rs1669691	upstream	*TDRP*	G	C	0.26	4.06(2.37, 6.97)	3.64E-07	3.89(2.29, 6.61)	5.36E-07
8	547,247/ 497,247	rs1669707	intergenic	***TDRP*** *-ERICH1*	C	G	0.26	4.02(2.34, 6.90)	4.30E-07	3.85(2.27, 6.55)	6.32E-07
8	68,931,007/ 69,843,242	rs6999003	ncRNA intronic	*LINC01592*	A	G	0.21	3.25(1.97, 5.36)	3.98E-06	3.13(1.91, 5.14)	5.94E-06
8	68,931,341/ 69,843,576	rs7463208	ncRNA intronic	*LINC01592*	G	A	0.30	3.00(1.87, 4.81)	5.37E-06	2.90(1.82, 4.62)	7.83E-06
8	68,932,173/ 69,844,408	rs7460431	ncRNA intronic	*LINC01592*	C	T	0.30	3.00(1.87, 4.81)	5.37E-06	2.90(1.82, 4.62)	7.83E-06
8	68,932,854/ 69,845,089	rs7464228	ncRNA intronic	*LINC01592*	A	C	0.30	3.00(1.87, 4.81)	5.37E-06	2.90(1.82, 4.62)	7.83E-06
8	68,941,406/ 69,853,641	rs66891172	ncRNA intronic	*LINC01592*	G	C	0.25	3.23(1.97, 5.31)	3.45E-06	3.12(1.92, 5.10)	5.07E-06
10	106,675,755/ 108,435,513	rs821932	intronic	*SORCS1*	A	G	0.19	3.15(1.91, 5.18)	6.29E-06	3.05(1.86, 4.99)	9.39E-06
18	3,423,021/ 3,423,019	rs73375993	intronic	*TGIF1*	G	A	0.07	5.95(2.74, 12.89)	6.27E-06	5.67(2.63, 12.22)	9.25E-06
18	3,554,526/ 3,554,524	rs7238797	intronic	*DLGAP1*	C	T	0.18	3.59(2.07, 6.21)	5.11E-06	3.46(2.01, 5.96)	7.26E-06
18	70,469,288/ 68,136,524	rs28505079	intergenic	***LINC01910*** *-GTSCR1*	A	G	0.31	2.89(1.82, 4.59)	6.71E-06	2.80(1.77, 4.41)	9.69E-06

*We included the SNPs if the P-value<1.0E-05 from both logistic and Firth regression models. The logistic and Firth regression model included *Ct* reinfection status as an outcome and SNPs as predictors with age, African ancestry, new sex partner, and unprotected sex since the last visit as covariates. Bold genes indicate the closest gene to the SNP.

### Replication analysis

Since a true replication sample for reinfection GWAS was unavailable, we performed available GWAS look-ups on chlamydia-related risk factors, including *Ct* susceptibility and chlamydia-related female infertility (38, 39). **Supplementary Table S4** showcases statistical significance in reinfection GWAS corresponding to significant SNPs in *Ct* susceptibility and chlamydia*-*related female infertility. We observed only one significant SNP, rs9304095 (*DSG4*), which had a P-value of 7.93E-04 in chlamydia reinfection GWAS and a corresponding P-value of 5.52E-07 in chlamydia-related infertility.

### Post-GWAS analyses

The main goal of the post-GWAS analysis is to prioritize the significant GWAS SNPs in protein-coding or non-protein-coding regions for their potential cellular/molecular/biological functions related to chlamydia reinfection for future investigations. **Supplementary Figure S3** depicts the strategy for prioritizing putative SNPs identified by GWAS.

### Linkage disequilibrium and the extent of significance near significant GWAS SNPs

**Figure 2:**
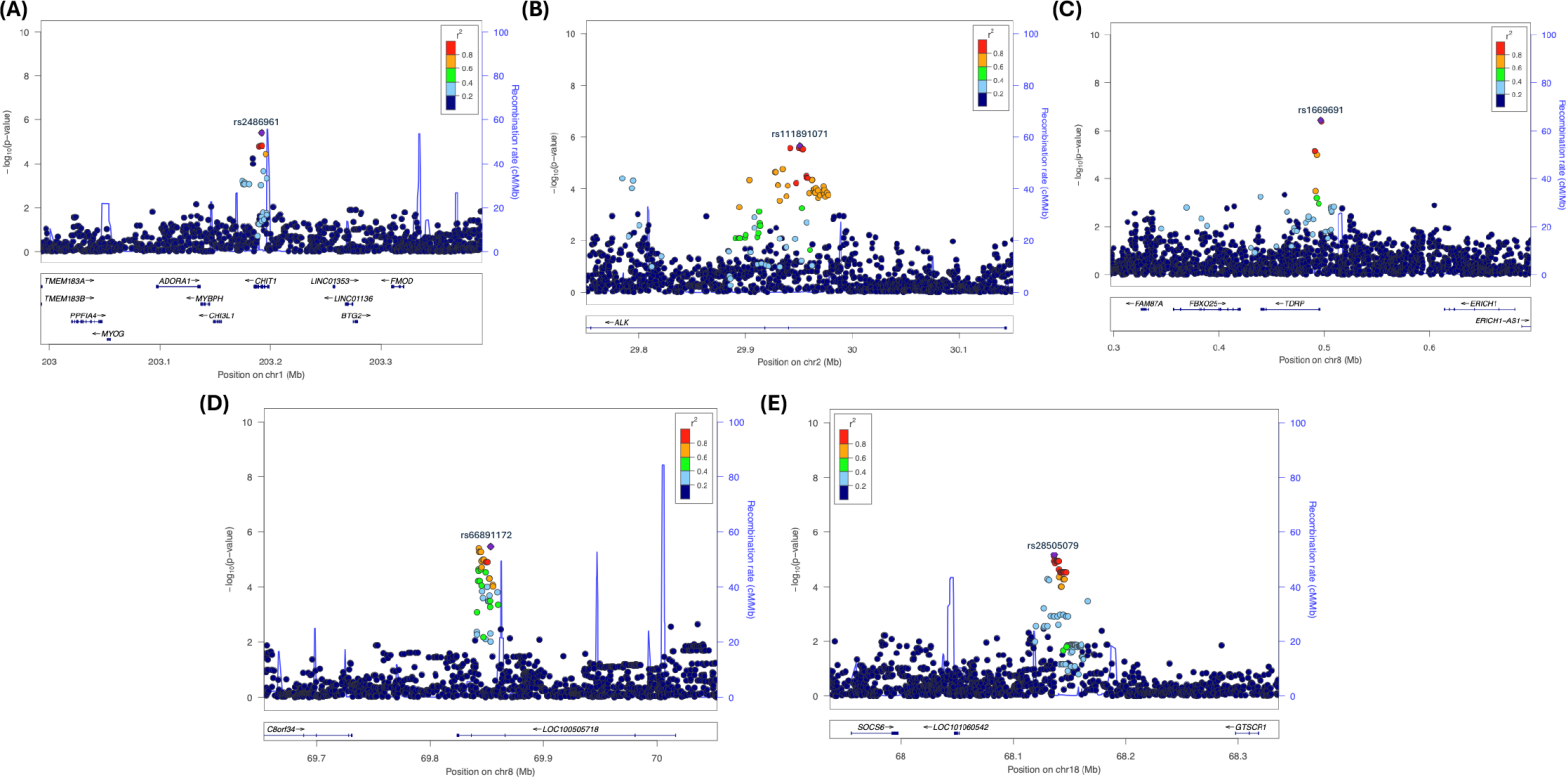
Locus Zoom plots showing strong LD with putative locus in Chromosomes 1, 2, 8, and 18. **(A)** rs24869x1 (Chr. 1, *CHIT1*); **(B)** rs77791547 (Chr. 2, *ALK*); **(C)** rsrs1669691 (Chr. 8, *TDRP*); **(D)** rs66891172 (Chr. 8, *LINC01592*); and **(E)** rs28505079 (Chr. 18, *LINC0190-GTSCR1*).

Note that the statistically significant GWAS SNP (sentinel SNP) does not indicate the SNP is causal. Other SNP(s) might be causal due to high correlation (i.e., strong linkage LD with the sentinel SNP within the haplotype block) (67–72). Also, note that the majority of GWAS findings (>90%) of disease/trait-associated SNPs are in non-protein-coding regions of the genome away from the known genes, suggesting that sentinel SNP or SNPs in strong LD might be affecting the disease risk by altering the gene regulation of one or more target gene expressions (69–75). We used LocusZoom plots to determine the strong LD support for the sentinel SNPs and the extent of significant SNPs near them. LocusZoom plots provide visual inspection of the significant SNP’s association and nearby SNPs’ association strength, as well as LD information between loci to determine the extent of the association signals and the position relative to nearby SNPs and genes, since genes several hundred kb from an associated significant SNP might be functionally relevant (76). There were only 5 sentinel SNPs that had strong LD support. The Locus-Zoom plots for selected SNPs in chromosomes 1, 2, 8 (2 SNPs), and 18 are shown in **Figure 2** with LD support. Other SNPs in **Table 1** did not exhibit any strong LD support. The Locus-Zoom plots for other SNPs with no apparent LD support are shown in **Supplementary Figure S4**.

### Fine mapping

**Figure 3:**
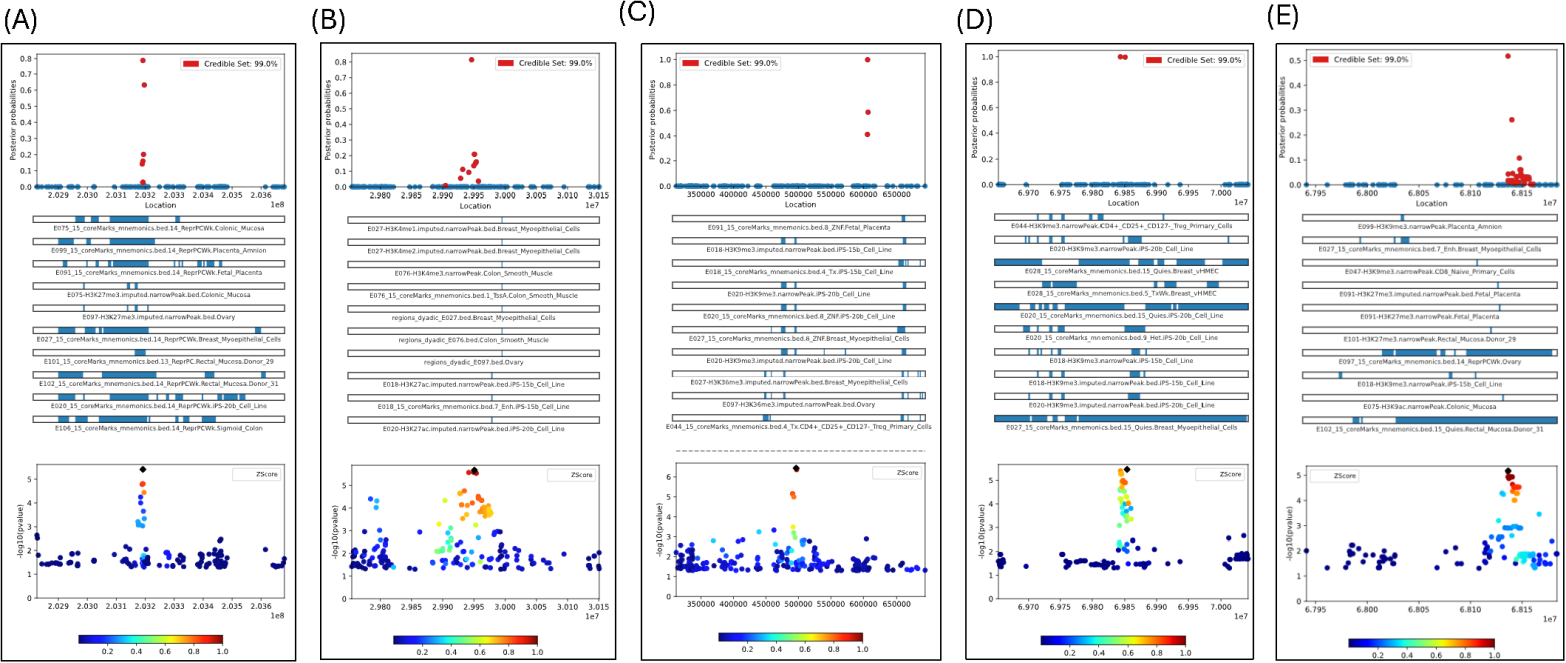
**(A)** Fine-mapping of *CHIT1* gene region (Chr1) with 163 SNPs with GWAS P-values<0.05 based on 1448 tracks; **(B)** Fine-mapping of *ALK* gene region (Chr2) with 263 SNPs with GWAS P-values <0.05 based on 1448 tracks; **(C)** Fine-mapping of TDRP gene region (Chr8) with 226 SNPs with GWAS P-values<0.05 based on 1448 tracks; **(D)** Fine-mapping of *LINC01592* gene region (Chr8) with 147 SNPs with GWAS P-values<0.05 based on 1448 tracks; Only plots included if posterior prob>0.8 **(E)** Fine-mapping of *LINCO1910-GTSCR1* gene region (Chr18) with 147 SNPs with GWAS P-values<0.05 based on 1448 tracks; Only plots included if posterior prob>0.8.

The next step was to fine-map the regions of interest found through LocusZoom to determine the possible causal SNPs for chlamydia reinfection, using PAINTOR. PAINTOR provides researchers with a short list of genetic variants from GWAS association results that are most likely to be causal. We performed fine mapping to find independent causal SNPs within the 5 regions identified by LocusZoom by the extent of significance and LD within a 200 +/- kb window, using PAINTOR. We used the “posterior probability (PP)” assigned to each variant within a genomic region, indicating how likely the SNP is to be the causal variant for chlamydia reinfection, with higher probabilities signifying a greater chance of being causal. The variants with the highest posterior probabilities within a credible interval are most likely to be causal, while low posterior probability variants are less likely to be causal. This method considers both the GWAS signal and functional annotations to prioritize variants located in the regions with known biological relevance. We used a 99% credible interval of SNPs to focus on a smaller set of potentially functional variants rather than analyzing all SNPs in a region. **Figures 3A–E** depict the fine mapping results for 5 genomic regions. **Supplementary Tables S5A–E** contain the distribution of posterior probabilities (PPs) corresponding to these 5 genomic regions and **Supplementary Tables S6A–E** contain the marginal significance estimates for each annotation, overall likelihood ratio test (LRT) estimate and corresponding P-value contributing to posterior probabilities of SNPs within the 5 regions.

We found several SNPs likely to have a causal effect for chlamydia reinfection, based on the posterior probabilities of causality produced by the PAINTOR. Specifically, rs2486961 (GWAS P-value =3.87E-06) in *CHIT1* had the highest PP of 78.84% and there were two more SNPs, namely, rs1417150 (GWAS P-value=2.10E-02, 4853 bp from rs2486961) and rs2486963 (GWAS P-value=3.59E-02, 2104 bp from rs2486961) had PP of 63.17% and 20.15%, respectively (see **Figure 3A**; **Supplementary Table S5A**). The top ten tissue annotation tracks contributing to the posterior probability of SNPs showing enrichment likelihood ratio test (LRT) P <0.05 were associated with 15-state chromatin marks from colonic mucosa, placenta amnion, fetal placenta, ovary, breast myoepithelial cells, rectal mucosa, B-cell lines, and sigmoid colon. Note that tissues involved in chlamydia reinfection mostly show strong regulatory functional potential (see **Supplementary Table S6A**). These results provide strong evidence that rs2487961 (*CHIT1*) may play a causal role in chlamydia reinfection and warrant further investigation in follow-up studies.

The most significant GWAS SNPs in the *ALK* gene did not have PP >0.80. Instead, rs13390546 (GWAS P-value =8.62E-03) in *ALK* had the highest posterior probability (PP) of 81.45% and there were two more SNPs, namely rs111891071 (GWAS P-value=2.13E-06, most significant in *ALK*, 4438 bp from rs13390546) and rs66953037 (GWAS P-value=3.59E-02, 2104 bp from rs13390546) had both PP of 20.85% (see **Figure 3B**; **Supplementary Table S5B**). The top ten tissue annotation tracks contributing to the posterior probability of SNPs showing enrichment LRT P <0.05 were associated with 15-state chromatin states from colon smooth muscle, breast myoepithelial cells, and ovary, and B-cell lines (see **Supplementary Table S6B**). The 15 different chromatin states are based on combinations of histone modifications in the genome. Chemical changes to DNA and histones (called epigenetic marks) affect whether a gene is turned on or off. The 15-state chromatin model helps interpret the regulatory landscape of the genome, which provides information about gene activity in different cells/tissues. These states help interpret the regulatory landscape of the genome for the Ct reinfection. Fine-mapping indicated that rs13390546 (*ALK*), rather than the significant GWAS SNP rs111891071, is likely the causal variant associated with chlamydia reinfection and associated with chromatin states from colon smooth muscle, breast myoepithelial cells, and ovary, B-cell lines.

We fine-mapped two regions on chromosome 8, namely one upstream of *TDRP* and another region within *LINC01592*. The most significant GWAS SNP rs1669691 (P = 3.64E-07) had PP = 0.001, implicating no causal effect; however, the SNP rs1703937 (109kb from rs1669691) in *TDRP* had PP = 99.84, showing strong potential for causal effect (see **Figure 3C**; **Supplementary Table S5C**). Two SNPs, rs4735900 (709 base pairs from rs1703937) and rs6996811 (252 base pairs from rs1703937), had PP of 58.65% and 41.19%, respectively (**Supplementary Table S5C**). The tissue annotation tracks contributing to the posterior probability of SNPs showing enrichment LRT P<0.05, were associated with 15-state chromatin marks from 49 different annotation tracks including B-cell lines, breast myoepithelial cells, ovary, fetal placenta, colon and rectal smooth muscle, colonic and rectal mucosa, sigmoid colon, breast mammary epithelial cells, several types of CD4+ T-cells, CD8 memory cells from cervical carcinoma, CD8 and CD4 primary cells (see **Supplementary Table S6C**). In the *LINC01592* region, SNP rs6998830 (GWAS P = 2.61E-05) had a PP of 100% and was 10kb from the most significant SNP rs6999003 (3.98E-06) in the region (see **Figure 3D**; **Supplementary Table S5D**). Also, rs4737926 (3029 base pairs from rs6999003) had a PP of 99.76% (**Supplementary Table S5D**). Both SNPs are strong candidates to be causal with high posterior probability. However, none of the annotation tracks were significant with LRT P <0.05 (**Supplementary Table S6D**). In summary, our findings suggest that rs1703937 (*TDRP*) and two SNPs (rs6998830 and rs4737926) in *LINC01592* are potentially causal variants.

In the chromosome 18 intergenic region, there were two SNPs with PP >0.2, namely, rs28373933 (PP = 51.74%; GWAS P = 7.49E-06; 141 base pairs from the most significant GWAS SNP rs28505079 [P-value=6.71E-06] in the region) and rs9965095 (PP = 26.15%; GWAS P = 2.49E-03; and 3,722 base pairs from rs28505079) (see **Figure 3E**; **Supplementary Table S5E**). The only annotation track H3K9me3 related to placenta amnion was significant with LRT P<0.05 (**Supplementary Table S6E**). The fine mapping identified a single intergenic SNP, rs28373933, with a posterior probability (PP) greater than 0.5, suggesting a potential causal link to chlamydia reinfection.

### GWAS SNPs enrichment

We used SNPs with P <1.0E-2 for SNP2GENE analysis. There were 16 genomic loci, 19 independent SNPs, and 17 lead SNPs containing chlamydia reinfection GWAS SNPs with P <1.00E-05 (**Supplementary Table S7**). The chlamydia reinfection GWAS SNPs enrichment statistics for functional consequences are provided in **Supplementary Table S8** and **Supplementary Figure S5**. Reinfection GWAS SNPs were enriched with intergenic (number of candidate SNPs = 69; proportion of candidate SNPs=30.3%; P = 1.43E-06), intronic (number of candidate SNPs = 105; proportion of candidate SNPs=46.1%; P = 4.77E-03), and ncRNA intronic (number of candidate SNPs = 49; proportion of candidate SNPs=21.5%; P = 1.42E-05).

### Gene-based test

FUMA implements MAGMA gene-based analysis using the GWAS input data. In the gene-based test, a few genes or nearby genes close to chlamydia reinfection GWAS SNPs were significant, e.g., genes *DLGAP1* (Chr. 18, P = 1.17E-10), *TDRP* (Chr. 8, P-value =1.20E-08), *CHI3L1* (Chr. 1, P = 8.77E-08), *CHIT1* (Chr. 1, P = 8.77E-08), *MYBPH* (Chr. 1, P = 1.80E-07), *UST* (Chr. 6, P = 6.22E-07), *FBXO25* (Chr.8; 8.41E-07), *DLGAP2* (Chr. 8, P = 8.46E-07), *TGIF1* (P = 1.42E-06), and *ALK* (Chr. 2, P = 2.83E-06) (**Supplementary Table S9**). In summary, gene-based analysis identified several significant genes near chlamydia reinfection-associated SNPs, including *DLGAP1*, *TDRP*, *CHI3L1*, *CHIT1*, *MYBPH*, *UST*, *FBXO25*, *DLGAP2*, *TGIF1*, and *ALK*. These findings highlight potential candidate genes for further investigation.

### Tissue-specific expression

MAGMA tissue-specific gene expression analysis results are given in **Supplementary Figure S6** and **Supplementary Tables S10a, b**, corresponding to 30 general tissues and 54 specific tissues, respectively. The gene expression on the fallopian tube (P = 9.00E-04) and adipose tissues (P = 1.11E-03) was statistically significant after Bonferroni correction in general tissues analysis (**Supplementary Table S10a**). In addition, the gene expressions on the uterus and cervix uteri were significant with a P <0.05 in general tissue analysis. We did not observe any statistically significant gene expression after Bonferroni correction in 54 specific tissues. However, the gene expressions on the fallopian tube (P = 1.24E-02), uterus (P = 3.63E-02), and endocervix (P = 5.79E-02) were marginally significant after Bonferroni correction (**Supplementary Table S10b**). In summary, the tissue-specific gene expression analysis revealed statistically significant associations in the fallopian tube and adipose tissue after Bonferroni correction in the general tissue analysis, with additional nominal significance in the uterus and cervix uteri.

### eQTL and chromatin interactions

The SNP2GENE module of FUMA also performs positional mapping, eQTLs, and CIs analyses. The summary information of positional mapping, eQTLs, and CIs analysis results is shown in **Table 2**. **Supplementary Table S11** provides more detailed information on FUMA SNP2GENE’s positional, eQTLs, and CI mapping of functionally relevant SNPs using chlamydia reinfection GWAS SNPs. **Supplementary Table S12** describes the strength of SNP-gene-tissue eQTLs found using reinfection overlapping GWAS SNPs, and **Supplementary Table S13** contains the trimmed version of significant intra-chromosomal CIs results from the SNP2GENE FUMA module. We included CIs if the gene had an ENSEMBL ID and was filtered for CI interaction. Furthermore, we also provide Hugo Gene Nomenclature (HGNC) gene symbols corresponding to ENSEMBL gene IDs since FUMA only provides ENSEMBL gene IDs involved in chromatin interactions. We used BioTools.fr (https://www.biotools.fr/human/ensembl_symbol_converter) to convert ENSEMBL gene IDs to HUGO gene symbols. Note that five ENSEMBL gene IDs in chromosome 1 did not have Hugo gene symbols, namely, ENSG00000272005; ENSG00000253640; ENSG00000237647; ENSG00000254269; and ENSG00000221446. We also investigated gene symbol converter using Ensembl Biomart ((https://useast.ensembl.org/biomart/martview/e763d80a463c64bbff071da94bb1f247), to convert Ensemble IDs to Hugo genes. Similar results were obtained.

**Table 2:** The summary information of positional mapping, eQTLs, and CIs analysis results using SNP2GENE module in FUMA.

Gene Symbol	Chr.	Start Position (build 37)	End Position (build 37)	Number of SNPs mapped to gene based on positional mapping	Number of SNPs mapped to gene based on eQTL mapping	The minimum eQTL P-value of mapped SNPs	The minimum eQTL FDR	eQTL Direction	Chromatin Interaction Mapping (Yes/No)	The minimum P-value of mapped SNPs	rsID of the independent significant SNPs that are in LD with the mapped SNPs
*CHIT1*	1	203181955	203242769	12	12	6.33E-49	0	–	Yes	3.87E-06	rs2486961
*ADORA1*	1	203059782	203136533	2	5	1.36E-06	5.21E-08	+	Yes	3.87E-06	rs2486961
*CHI3L1*	1	203148059	203155877	12	1	9.48E-06	2.68E-02	NA	Yes	3.87E-06	rs2486961
*RP11-569A11.1*	1	202573396	202574421	0	1	5.28E-04	3.46E-02	NA	No	NA	rs2486961
*TDRP*	8	439803	495781	10	10	4.38E-32	1.94E-25	–	No	3.64E-07	rs1669691
*RP11-91J19.3*	8	400714	401343	0	9	6.61E-09	3.23E-05	+	Yes	3.64E-07	rs1669691
*FBXO25*	8	356428	421225	0	5	1.23E-05	2.55E-04	+	No	6.98E-06	rs1669691
*FAM87A*	8	325931	333174	0	1	3.29E-04	2.34E-02	+	No	0.00109	rs1669691
*SULF1*	8	70378859	70573150	0	21	2.10E-09	4.29E-07	+	Yes	3.45E-06	rs66891172 rs6999003
*RP11-403D15.2*	8	68994439	69007316	0	9	1.99E-04	1.61E-02	+	No	3.98E-06	rs6999003
*SOCS6*	18	67956137	67997436	0	52	1.26E-06	8.07E-22	+	No	6.71E-06	rs28505079
*RP11-529J17.1*	18	68696006	68700292	0	1	7.84E-04	4.76E-02	NA	No	NA	rs28505079

**Figure 4:**
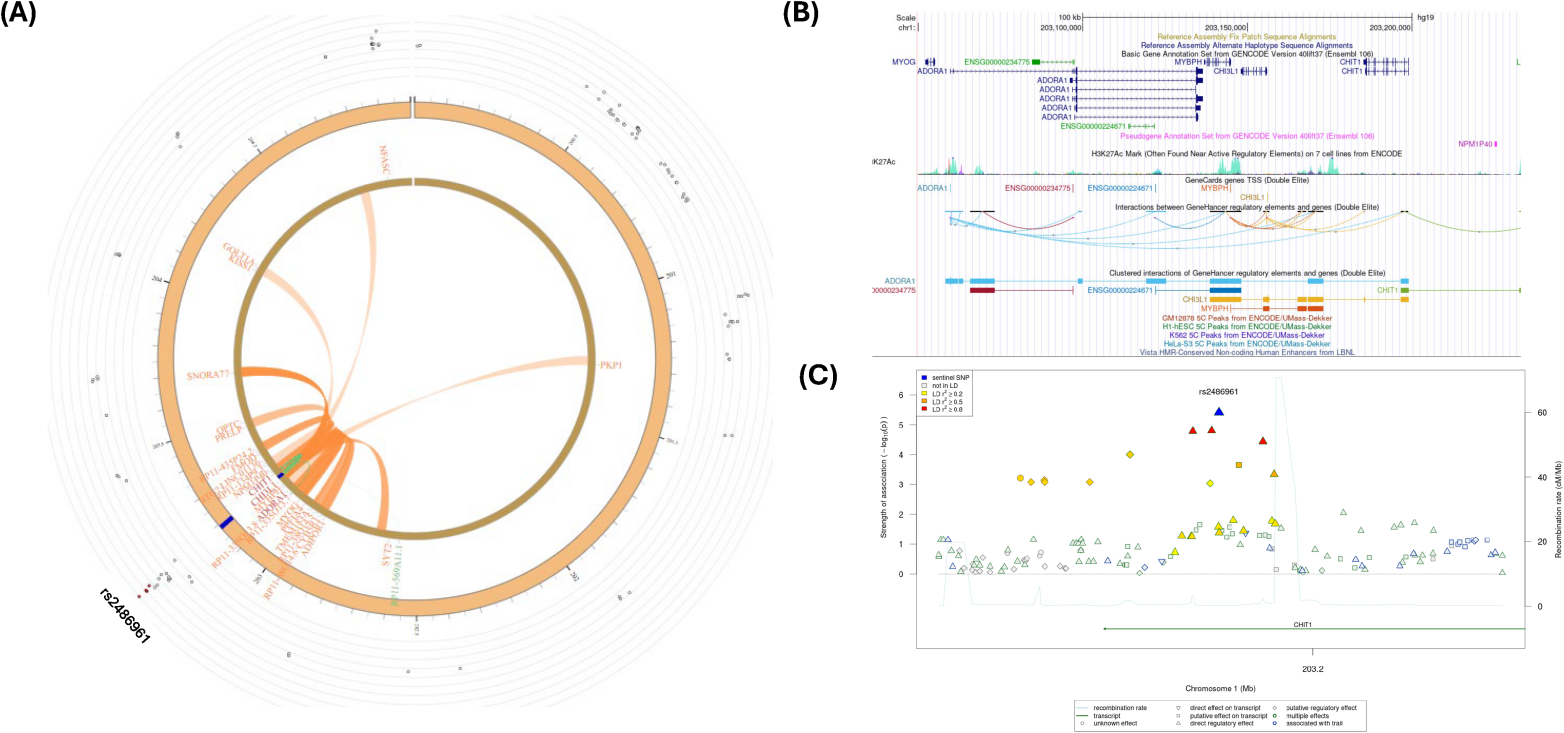
**(A)** Circos plot generated in FUMA showing various levels of information at rs2486961 locus in chromosome 1. The outer layer is the GWAS P- value for the SNP rs2486961, orange band with darker blue indicating identified risk loci, next are genes with known chromatin interactions with variants in orange, eQTLs are in green, and genes in red have evidence for both eQTL and chromatin interactions with the variant. **(B)** Annotation of GWAS variant rs2486961 (near *CHIT1*) using UCSC browser GrCh38 with regulatory marks in the region, known chromatin interactions; **(C)** SNIPA regional association plot with regulatory potential near the variant rs2486961.

**Figure 4A** depicts a Circos plot (77) generated in FUMA for SNP rs2486961, **Figure 4B** contains annotation of the GWAS variant rs2486961 (near *CHIT1*) using the Human UCSC browser GrCh38 with regulatory marks in the region, known as CIs, and **Figure 4C** shows the regulatory potential near the variant rs2486961 using SNiPA software (78). The eQTL analyses showed 4 significant eQTLs at the FDR level of 0.05, namely, *CHIT1*, *CHI3L1*, *ADORA1, and SYT2-AS1* (*RP11-569A11.1*) genes (**Table 2**; **Supplementary Tables S11**, **S12**). In addition, CIs were observed with SNPs in the candidate region (Chr.1:203190000-203200000) containing rs2486961 and with several genes in other genomic regions (**Supplementary Table S13**). SNP rs2486961 in chromosome 1 showed both CIs and eQTL with Chitinase 1 (*CHIT1*), Chitinase-3-like-1 (*CHI3L1*), and Adenosine A1 Receptor (*ADORA1*) genes (**Table 2**; **Supplementary Tables S11**–**S13**). **Figure 4B** shows potential interactions of GeneHancer regulatory elements and the *CHIT1* gene and the presence of the H3K27Ac mark. Furthermore, **Figure 4C** showcases 3 regulatory SNPs in linkage disequilibrium with rs2486961 using SNiPA. Note that SNiPA uses hg19 data to plot regulatory SNPs near the index SNP. In summary, eQTL and CI analyses revealed significant regulatory associations of rs2486961 with *CHIT1, CHI3L1, ADORA1*, and *SYT2-AS1*, highlighting its potential role in gene regulation within and beyond the candidate region.

**Figure 5:**
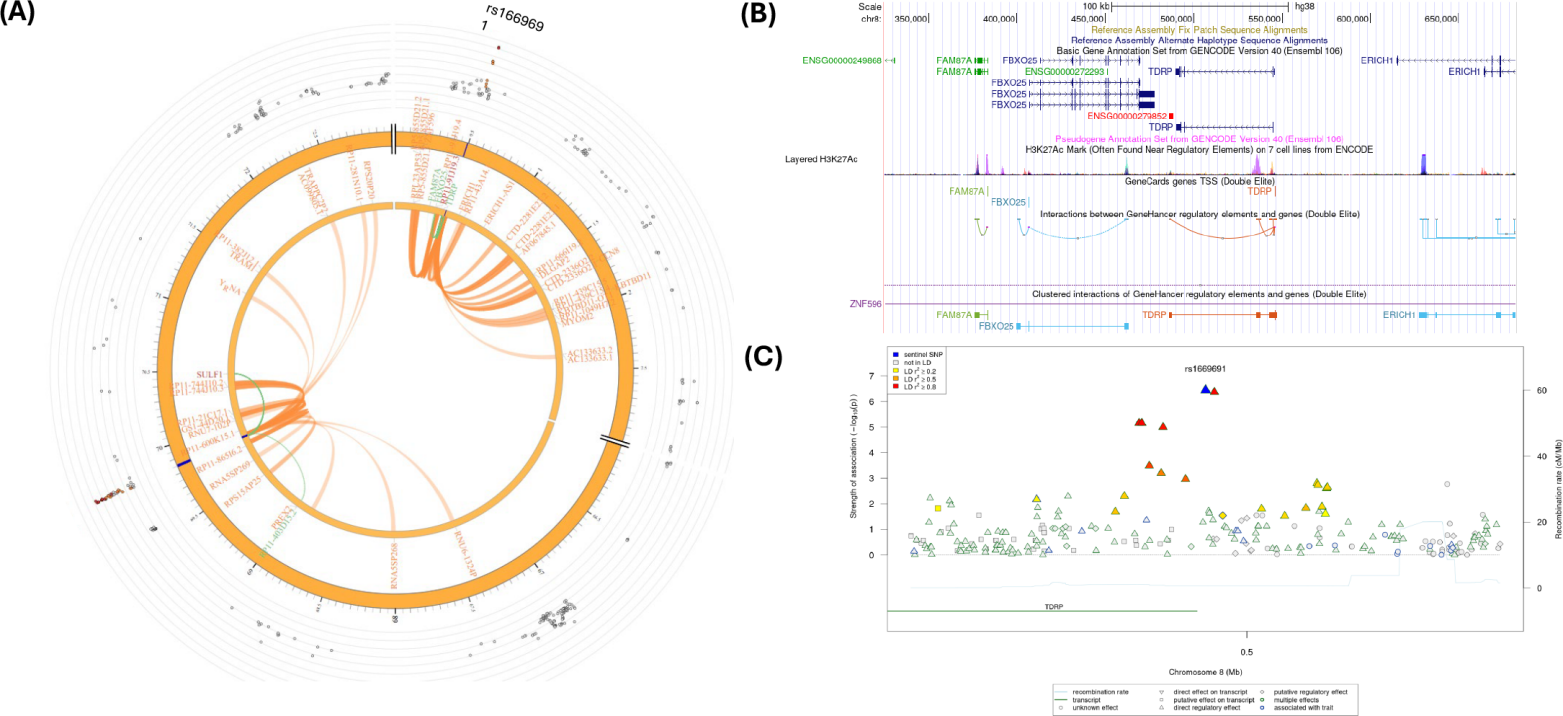
**(A)** Circos plot generated in FUMA showing various levels of information at rs1669691 locus in chromosome 8. The outer layer is the GWAS P- value for the SNP rs1669691, orange band with darker blue indicating identified risk loci, next are genes with known chromatin interactions with variants in orange, eQTLs are in green, and genes in red have evidence for both eQTL and chromatin interactions with the variant; **(B)** Annotation of GWAS variant rs 1669691 near *TDRP* gene using UCSC browser GrCh38 with regulatory marks in the region, known chromatin interactions; **(C)** SNIPA regional association plot with regulatory potential near the variant rs1669691.

**Figure 5A** shows a Circos plot generated by FUMA for SNP rs1669691 in chromosome 8. The eQTL analyses showed 4 significant eQTLs at the FDR level of 0.05, namely, *TDRP*, *RP11-91J19.3*, *FBXO25, and FAM87A* genes (**Table 2**; **Supplementary Tables S11**, **S12**). In addition, several CIs were observed with SNPs in the candidate region (Chr.8:480,001-520,000) containing rs1669691 and with several genes in other genomic regions (**Supplementary Table S13**). rs1669691 showed significant CI and eQTL target with *RP11-91J19.3* (ENSG00000272293) gene (**Table 2** and **Supplementary Tables S11**, **S12**, **S13**). **Figure 5B** shows potential interactions of GeneHancer regulatory elements and *TDRP*, *FBXO25*, and *FAM87A*. Furthermore, **Figure 5C** shows 7 regulatory SNPs in linkage disequilibrium with rs1669691 using SNiPA with r^2^>=0.5. In summary, rs1669691 demonstrates strong regulatory potential through its eQTL and CI associations with *TDRP*, *RP11-91J19.3*, *FBXO25, and FAM87A*.

**Figure 6:**
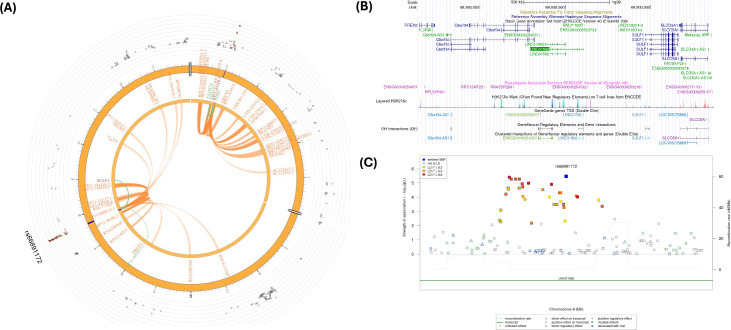
**(A)** Circos plot generated in FUMA showing various levels of information at rs66891172 locus in chromosome 8. The outer layer is the GWAS P- value for the SNP rs66891172, orange band with darker blue indicating identified risk loci, next are genes with known chromatin interactions with variants in orange, eQTLs are in green, and genes in red have evidence for both eQTL and chromatin interactions with the variant; **(B)** Annotation of GWAS variant rs66891172 near *LINC01592* using UCSC browser GrCh38 with regulatory marks in the region, known chromatin interactions; **(C)** SNIPA regional association plot with regulatory potential near the variant rs66891172.

SNP rs66891172 (*LINC01592*) had significant CI and eQTL (**Table 2**; **Figure 6A**) with the *SULF1* gene. In addition, the *RP11-403D15.2* gene was involved in eQTL mapping, but not in CI (**Table 2**; **Supplementary Tables S11**–**S13**). However, several CIs were observed with SNPs in the candidate region (Chr. 8:698,400,01-698,800,00) containing rs66891172 and with several genes in other genomic regions (**Supplementary Table S13**). **Figure 6B** shows several H3K27ac marks near the rs66891172. There were several potential regulatory SNPs in LD with rs66891172 (**Figure 6C**).

**Figure 7:**
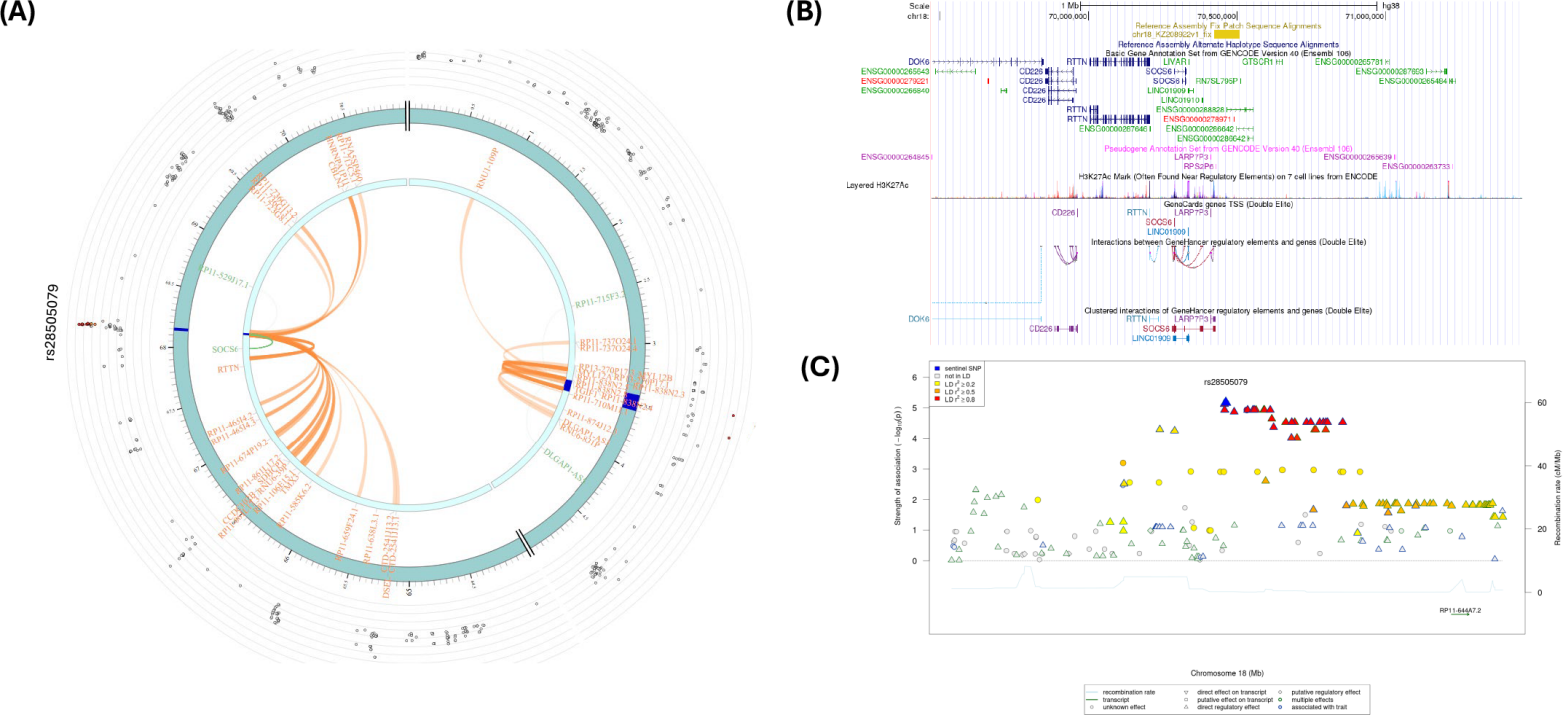
**(A)** Circos plot generated in FUMA showing various levels of information at rs28505079 locus in chromosome 18. The outer layer is the GWAS P-value for the SNP rs28505079, orange band with darker blue indicating identified risk loci, next are genes with known chromatin interactions with variants in orange, eQTLs are in green, and genes in red have evidence for both eQTL and chromatin interactions with the variant; **(B)** Annotation of GWAS variant rs28505079 using UCSC browser GrCh38 with regulatory marks in the region, known chromatin interactions; **(C)** SNIPA regional association plot with regulatory potential near the variant rs28505079.

The intergenic SNP rs28505079 (*LINC01910-GTSCR1*) had significant eQTL at *SOCS6* (**Table 2**; **Figure 7A**; **Supplementary Tables S11**, **S12**). In addition, several CIs were observed with SNPs in the candidate region (Chr. 18: 681,200,01-681,600,00) containing rs28505079 and genes in other genomic regions (**Supplementary Table S13**). **Figure 7B** shows several H3K27ac marks near the rs28505079, showing several potential chromatin interactions. In addition, there were several potential regulatory SNPs in LD with rs28505079 (**Figure 7C**). SNP rs28505079, located between *LINC01910* and *GTSCR1*, showed a significant eQTL with *SOCS6* and multiple chromatin interactions (CIs) within its candidate region and with distant genes.

FUMA also provided chromatin interactions in other chromosomes, specifically on chromosomes 2, 5, 6, 10, 16, and 20, and they are included in **Supplementary Figure S7**.

### Gene-set analysis

Gene-set and pathway/functional enrichment analysis provides biological context on how genes interact with each other, contributing to a larger biological process and providing insights into the underlying mechanisms of a disease, unlike analyzing single genes in isolation (79–82). The gene-set analysis using MAGMA gene-set in FUMA resulted in 10 gene-sets with P-value<1.0E-05, including NEGATIVE_REGULATION_OF_CYTOKINESIS (Bonferroni P = 4.21E-03), EMBRYONIC_CARCINOMA_DN (Bonferroni P = 5.96E-03), DEVELOPMENTAL_PROCESS_INVOLVED_IN_REPRODUCTION (Bonferroni P = 4.98E-02), CHORIONIC_TROPHOBLAST_CELL_DIFFERENTIATION (Bonferroni P = 5.56E-02), EXTRAEMBRYONIC_MEMBRANE_DEVELOPMENT (Bonferroni P = 6.63E-02) (**Supplementary Table S14**). Note that chlamydia is a known cause of infertility, and reinfections contribute to heightened infertility risk. In summary, the most notable pathways involved reproductive and developmental processes, aligning with chlamydia’s established association with infertility, while the enrichment of the negative regulation of the cytokinesis pathway suggests a potential role in the immune response to chlamydia infection.

### Overlapping GWAS

FUMA provided overlapping known GWAS SNPs corresponding SNPs from chlamydia reinfection GWAS and is given in **Supplementary Table S15**. rs2486961 and rs28505079 were associated with cerebrospinal fluid biomarker levels and blood protein levels, respectively.

### Single cell type analysis

FUMA uses GWAS genes for all scRNA-seq data to determine gene expression in cell type analyses. We performed cell type specificity analysis in FUMA corresponding to GWAS SNPs with P-value<10^-2^. **Supplementary Tables S16**, **S17** showcase cell type specificity analyses in the ovary and uterus, respectively, with scRNA-seq in the Mouse Cell Atlas. Furthermore, **Supplementary Figures S8A, B** depict cell type specificity with scRNA-seq data sets in FUMA using MAGMA gene property analysis. (A) shows significant cell types across datasets-Step1; (B) shows independent cell type associations based on within-dataset conditional analyses (Step 2). The results for cell type specificity analyses showing significant cell types across data sets (step 1) and independent cell type associations based on within-dataset conditional analyses (Step 2) are given in **Supplementary Tables S18**, **S19**, respectively. Note that stromal cells showed significant association with GWAS SNPs in the Mouse Atlas data set in both Steps 1 and 2. In particular, Stromal_cell_Has1_high and Stromal_cell_Cd111_high were significant with Bonferroni correction. We also performed PCGA analysis of cell types related to women’s reproductive system tissues, and the results of the analysis are given in **Supplementary Table S20**. Genes associated with GWAS SNPs were significantly expressed on Adult-Cervix stromal cells, indicating a high number of lymphocytes present within the stromal tissue of the cervix and Adult-Uterus1.Fibroblast expression in human data sets (**Supplementary Figure S9**). FUMA’s cell type specificity analysis, using GWAS-significant genes and scRNA-seq data, revealed significant associations in reproductive tissues, particularly in the ovary and uterus. Notably, the stromal cells from the Mouse Cell Atlas. Additional analysis using PCGA human single-cell data showed significant expression of GWAS-associated genes in Adult-Cervix stromal cells and Adult-Uterus fibroblasts, suggesting immune involvement and fibroblast activity in reproductive tissues.

### VEGAS2 gene-based and pathway analysis

In addition, we performed pathway analysis. We used SNPs with P <1.0E-02 for the gene-based test and pathway analysis using VEGAS2Pathway software. There were 8,059 genes with P < 1.0E-02. With Bonferroni correction, the P-value threshold for significance is 6.20E-06. Adjusting for 8,059 gene-based tests, 11 genes were significant with Bonferroni correction, including *CHI3L1*, *CHIT1*, and *MYBPH* on chromosome 1; *ALK* on chromosome 2; *UST* and *LOC100422737* on chromosome 6, *TDRP* and *LOC100505718* on Chr 8; and *DLGAP1*, *DLGAP-AS1*, and *DLGAP-AS2* on chromosome 18 (**Supplementary Table S21**). The top five pathways were GO:0000003_reproduction, GO:0002376_immune_system_process, GO:0003700_transcription_factor_activity, GO:0004672_protein_kinase_activity, and GO:0005102_receptor_binding (**Supplementary Table S22**). In summary, Pathway analysis using VEGAS2Pathway identified 11 significant genes after Bonferroni correction, including notable genes such as *CHI3L1, CHIT1, MYBPH, ALK, UST, and TDRP*. The top enriched pathways included reproduction, immune system processes, transcription factor activity, protein kinase activity, and receptor binding, highlighting key biological processes potentially involved in chlamydia reinfection.

## Discussion

We performed the first GWAS on chlamydia reinfection study in AA women that revealed several associations of novel SNPs and genes with reinfection. The majority of the genes identified in reinfection were related to immune response. GWAS revealed 17 genetic loci associated with reinfection. In post-GWAS analyses, 5 putative loci were identified using LD and the extent of SNP significance in the genomic regions. Fine mapping of the 5 regions revealed several potentially causal non-exonic SNPs (4 intronic SNPs and 1 intergenic SNP), indicating the possible regulatory effect of SNPs in chlamydia reinfection. These five SNPs are likely to have a strong causal effect for chlamydia reinfection, based on the posterior probabilities (PPs) of causality. Specifically, rs2486961 in *CHIT1* had a PP of 79%; rs13390546 in *ALK* had a PP of 81%; rs1703937 upstream of *TDRP* had a PP of 100%, SNPs rs6998830 and rs4737926 in the *LINC01592* region had a PP of 100%. Intergenic SNP rs28373933 in the genomic region (*LINC01910-GTSCR1*) had a PP of 52%.

Note that all SNPs listed above were involved in CI/eQTL mapping except for SNPs in the anaplastic lymphoma kinase (*ALK)* gene. The *ALK* gene produces a protein involved in cell growth (83, 84). This gene encodes a receptor tyrosine kinase, which belongs to the insulin receptor superfamily that transmits signals from the cell surface into the cell. These signals are important for cell growth, division, and maturation and play a pivotal role in cellular communication needed in response to bacterial infection (83, 84). *Ct* induces Akt phosphorylation throughout its entire developmental life cycle and recruits phosphorylated Akt (pAkt) to the inclusion membrane (85). There are several pathways and interactions for the *ALK* gene relevant to chlamydia reinfection, including the immune checkpoint signaling pathway, ERK signaling, MAPK signaling, AKT signaling, JAK-STAT pathway, and infectious disease-related tyrosine kinases/adaptors, signal and transduction (83, 84).

It is well established that CIs regulate gene expression by bringing distal regulatory elements, such as super-enhancers, into close spatial proximity with promoters. Bacterial survival depends on shaping the host’s transcriptional signature, a process regulated at the chromatin level. Chromatin modification on histone proteins or DNA are common targets in response to bacteria in the host. Also, the eQTLs are important in understanding the biology of the significant GWAS genetic variants since they identify the variant involvement in the expression of the genes. Thus, both CI and eQTL are important in understanding the biology/potential mechanisms of the significant GWAS genetic variants in manifesting the disease/traits. We have identified several potential SNPs involved in chlamydia reinfection due to eQTLs and/or CIs mapping in *silico*. Specifically, we found 4 strong candidate SNPs, namely, rs2486961 (intronic, *CHIT1*, chr. 1), rs1669691 (upstream of *TDRP*), rs66891172 (ncRNA, *LINK01592*, Chr.8), and rs28505079 (intergenic, *LINC01910-GTSCR1*chr.18) with CIs/eQTL presence.

The rs2486961 has both CIs and eQTL presence with *CHIT1*, *CHI3L1*, and *ADORA1* genes. The *CHIT1* gene encodes plasma methylumbelliferyl tetra-N-acetylchitotetraoside hydrolase (chitotriosidase), a human chitinase enzyme (EC 3.2.1.14) (86–88). Chitotriosidase belongs to the family of 18 glycosyl hydrolases and was first discovered in the plasma of Gaucher disease patients (89). Chitinases and chitinase-like proteins are primarily expressed and secreted by phagocytes, mainly neutrophils and macrophages, and induced at sites of inflammation, infection, and tissue remodeling (88). Hydrolase activity plays a crucial role in bacterial infection because the host immune system utilizes hydrolases like lysozyme, which targets the bacterial cell wall and is a key component of the innate immune response against bacterial infections (90–92). *CHI3L1* is a pro-inflammatory cytokine that responds to other pro-inflammatory cytokines, such as TNF-α, interleukin-1β (IL1- β), interleukin-6 (IL-6), and IFN- γ) (93, 94). In 2018, Lee showed that the cytokine CHI3L1(YKL40) was significantly and positively associated with chlamydia cervical burden (P = 4.88E-04) and was also associated with endometrial chlamydial infection (P = 0.044), however, there was no association with endometrial chlamydial Infection observed after adjusting for oral contraceptive use, gonorrhea coinfection, and cervical chlamydial load (95, 96). Nevertheless, *CHI3L1* is associated with inflammation and tissue remodeling, common responses to infections, including chlamydia reinfection (95, 96). The *ADORA1* (Adenosine A1 Receptor) protein is an adenosine receptor belonging to the G-protein-coupled receptor 1 family (84, 97). *ADORA1* adenosine receptors are coupled to adenylyl cyclase via the inhibitory G-protein subunit (Ga_i_), which can reduce intracellular levels of the cyclic adenosine monophosphate (cAMP) (98). Note that millimolar concentrations of cAMP inhibit chlamydial development (99–101). The activation of *ADORA1* may decrease inflammation and apoptosis in chlamydia infection.

There were 4 genes identified as a target for rs1669691, namely, *TDRP*, *RP11-91J19.3*, *FBXO25*, and *FAM87A*, using eQTL and CI mapping. *FBOX25* was the strongest candidate gene for chlamydia reinfection. F-box proteins are one of the four subunits of the ubiquitin protein ligase complex known as SCFs (SKP1-cullin-F-box), which play a key role in phosphorylation-dependent ubiquitination (84, 102). *Chlamydia* manipulates the host cell’s actin cytoskeleton to establish itself and replicate (103–107). *Chlamydia* has evolved strategies to evade this immune response by producing proteins with deubiquitinating activity, removing the ubiquitin tags, and allowing the bacteria to survive within the host cell (108–111). On the other hand, *TDRP* gene SNPs are associated with IL-4 levels (112, 113). IL-4 is known to prevent tissue damage caused by excessive Th1 immune responses IL-4-secreting eosinophils promote the proliferation of endometrial stromal cells, helping to prevent chlamydia-induced damage to the upper genital tract (114–116). The role of *TDRP* in chlamydia infection or reinfection in women is not known; however, *TDRP* is expressed in women’s reproductive system ordered by median TPM (transcripts per million) values from high to low (ovary, fallopian tube, endocervix, uterus, vagina, and ectocervix) (117). There is very little known about the relationship between chlamydia and *RP11.91J19.3* and *FAM87A* genes.

*LINC01592* (Long Intergenic Non-Protein Coding RNA 1592) in chromosome 8 is an RNA Gene. There is not much known about *LINC01592*’s role in response to chlamydia infection or reinfection. However, *LINC01592* is known to suppress the immune system, facilitating MHC-I degradation through the autophagy-lysosome pathway in esophageal cancer cells to evade detection by cytotoxic T cells (118). Chlamydiae reside in host cells within a vacuole known as an inclusion. To replicate, chlamydiae need nutrients and membranes for the growth of inclusion (119). Autophagy is known to restrict bacterial proliferation in several bacterial diseases such as *Legionella*, *Salmonella*, and mycobacterium infections, reducing the infection’s severity and dissemination (120–122). Conflicting results have been reported regarding the role of autophagy in *Ct* proliferation, with outcomes varying based on experimental conditions, chlamydial serovars, and cell lines used (107, 120). As it is well known that nutrient availability impacts *Ct* proliferation in host cytoplasm, autophagy may serve as a nutrient source for *Ct* replication (120). In addition, *LINC01592* is highly expressed in the ectocervix and endocervix, vagina, uterus, and fallopian tube.

Also, SNP rs66891172 in the *LINC01592* (Long Intergenic Non-Protein Coding RNA 1592) is involved as an eQTL with the *SULF1* gene. The *SULF1* gene encodes an extracellular heparan sulfate endosulfatases (84, 123). *SULF1* is known to exhibit arylsulfatase activity and highly specific endoglucosamine-6-sulfatase activity and also can remove 6-O-sulfate groups from heparan sulfate chains of heparan sulfate proteoglycans (HSPGs) (84, 123–125). The *SULF1* is highly expressed in the endometrium and fallopian tubes. Endosulfatases *SULF1* and *SULF2* (Chr. 12) limit *Chlamydia muridarum* infection (126). Kim et al. showed that ectopic expression of *SULF1* or *SULF2* in HeLa cells decreased cell surface HSPG sulfation diminished *C. muridarum* binding and decreased vacuole formation (126). The *SULF1* gene is a strong candidate gene for chlamydia reinfection with a protective effect.

We also found intergenic SNP rs28505079 in *LINC01910* - *GTSCR1* in chromosome 18 as a candidate for eQTL with the *SOCS6* gene. The *SOCS6* gene is a part of the suppressor of cytokine signaling (SOCS) gene family. The protein encoded by *SOCS6* contains an SH2 domain and a CIS homolog domain, classifying it within the cytokine-induced STAT inhibitor (CIS) family, also known as the SOCS or STAT-induced STAT inhibitor (SSI) protein family (84, 127). CIS family members are recognized as cytokine-inducible negative regulators of cytokine signaling (127). SOCS family proteins are involved in a classical negative feedback system that regulates cytokine signal transduction. Additionally, they may also function as substrate recognition components of an SCF-like ECS (Elongin BC-CUL2/5-SOCS-box protein) E3 ubiquitin-protein ligase complex, mediating the ubiquitination and subsequent proteasomal degradation of target proteins (128). An SCF-like ECS E3 ubiquitin-protein ligase complex plays a crucial role in the bacterial infection response by regulating the degradation of key signaling proteins through ubiquitination, which is particularly important in controlling inflammatory pathways and immune cell responses to bacterial invasion (129, 130). *SOCS6* also regulates KIT receptor signaling degradation by ubiquitination of the tyrosine-phosphorylated receptor (131, 132). The KIT receptor signaling pathway plays a crucial role in the host immune response to bacterial infection, primarily by regulating the function of mast cells, which are important immune cells involved in inflammation and tissue repair when activated by binding to its ligand, Stem Cell Factor (SCF); however, excessive KIT signaling can also contribute to an uncontrolled inflammatory response during severe infections (133–135).

The main limitations of this study are the lack of true validation in chlamydia reinfection cohorts and the small sample size. Note that this study was the largest GWAS on chlamydia reinfection in a minority population. Further validations in different cohorts, as well as functional studies of the identified putative variants and genes, are warranted in the future. This study was also limited in that the chlamydia treatment used at the enrollment visit was azithromycin 1g single dose, which was a first-line CDC-recommended treatment at the time the study was conducted (9). While azithromycin is highly effective for urogenital chlamydia in women, it has lower cure rates for rectal chlamydia (136). Since women with urogenital chlamydia can have concomitant rectal chlamydia, which in theory could reinfect the urogenital site (136), it is possible some of the women with urogenital reinfection were infected from their rectal site rather than acquiring *Ct* infection at their urogenital site from sexual activity with a sexual partner; rectal swabs were not collected in this cohort, so we could not evaluate rectal chlamydia in women who were versus were not classified as having urogenital reinfection.

In conclusion, we found several strong candidate genes for chlamydia reinfection, e.g., *CHIT1, CHI3L1, ADORA1, ALK, TDRP, FBXO25, LINC01592, SULF1*, and *SOCS6*, involved in the immune response. *CHIT1, ADORA1, FBXO25, SULF1*, and *SOCS6* were identified due to CI/eQTL analyses with GWAS top hits showing possible mechanisms of chlamydia reinfection. The genes identified in this chlamydia reinfection GWAS study could be used for genetic testing to predict reinfection risk among women who may require more frequent *Ct* testing, which could directly benefit individual women as well as advance chlamydia prevention and control efforts. Additionally, these gene findings could guide future research into immune responses and mechanisms involved in chlamydia infection, which would guide to advance vaccine development efforts.

## Data Availability

The data presented in the study are deposited in the dbGaP repository, accession number phs004338.v1.p1.
