# Supplementary material for: Genome-wide association study of chlamydia reinfection in African American women
Source: Front Immunol. 2025 Sep 25;16:1594317. doi: 10.3389/fimmu.2025.1594317 (PMC12508776; doi:10.3389/fimmu.2025.1594317)
Supplement: Supplementary file 2 [file Presentation1.pptx]

## Slide 1
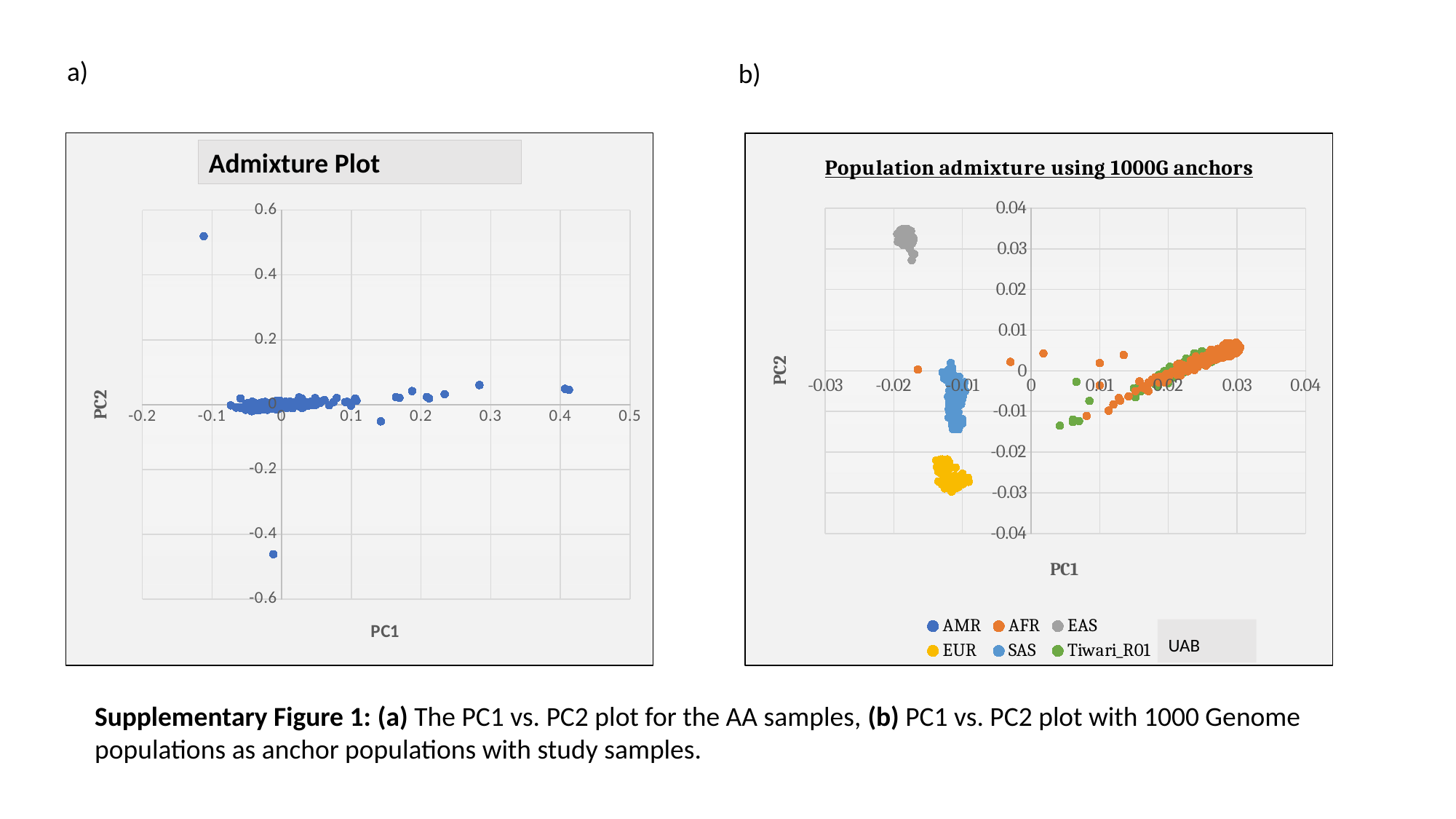

a)
b)
### Chart: Tiwari R01 admixture plot
| Category | |
|---|---|
### Chart: Population admixture using 1000G anchors
| Category | | | | | | |
|---|---|---|---|---|---|---|Admixture Plot
UAB
Supplementary Figure 1: (a) The PC1 vs. PC2 plot for the AA samples, (b) PC1 vs. PC2 plot with 1000 Genome populations as anchor populations with study samples.

## Slide 2
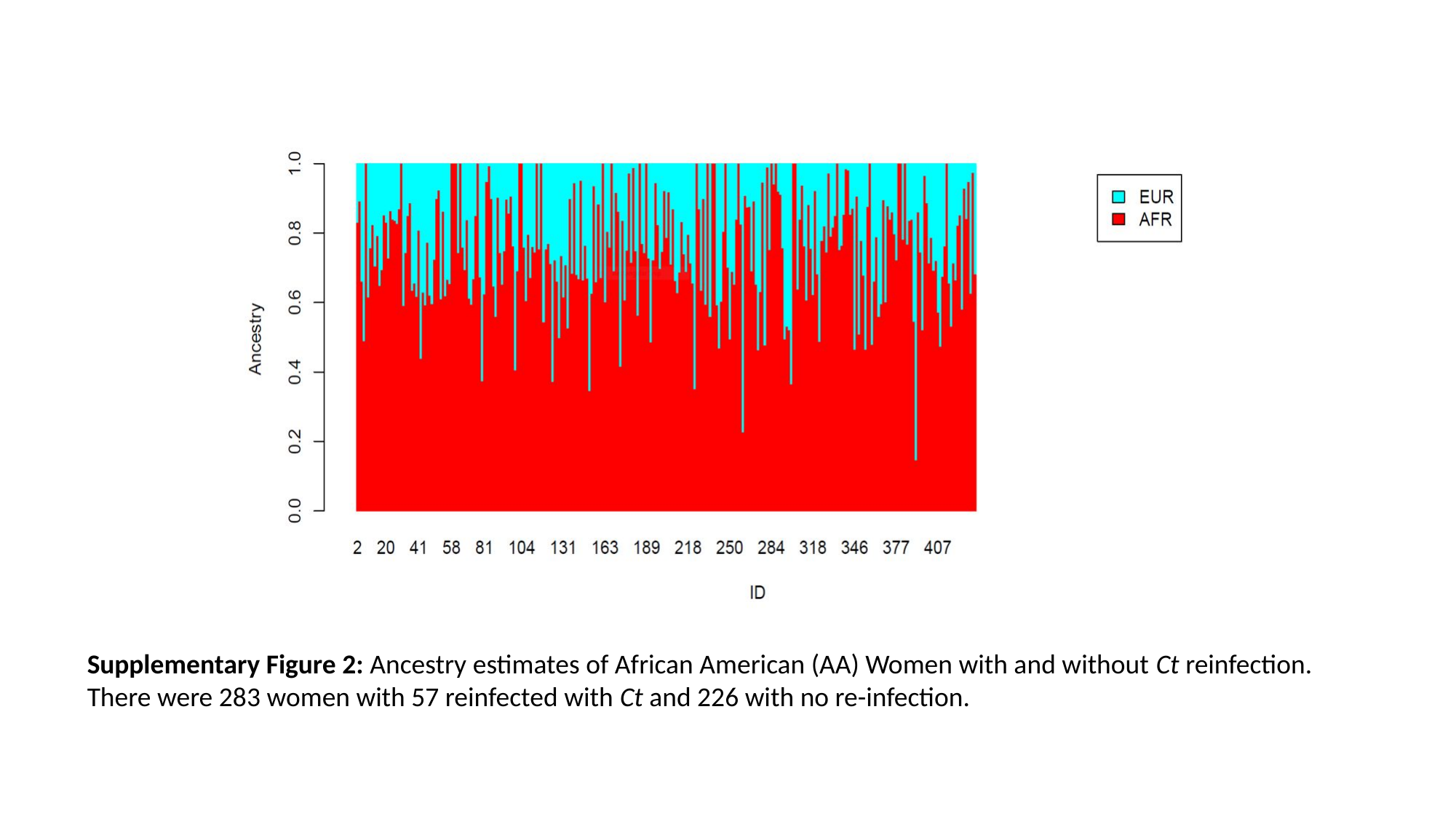

Supplementary Figure 2: Ancestry estimates of African American (AA) Women with and without Ct reinfection. There were 283 women with 57 reinfected with Ct and 226 with no re-infection.

## Slide 3
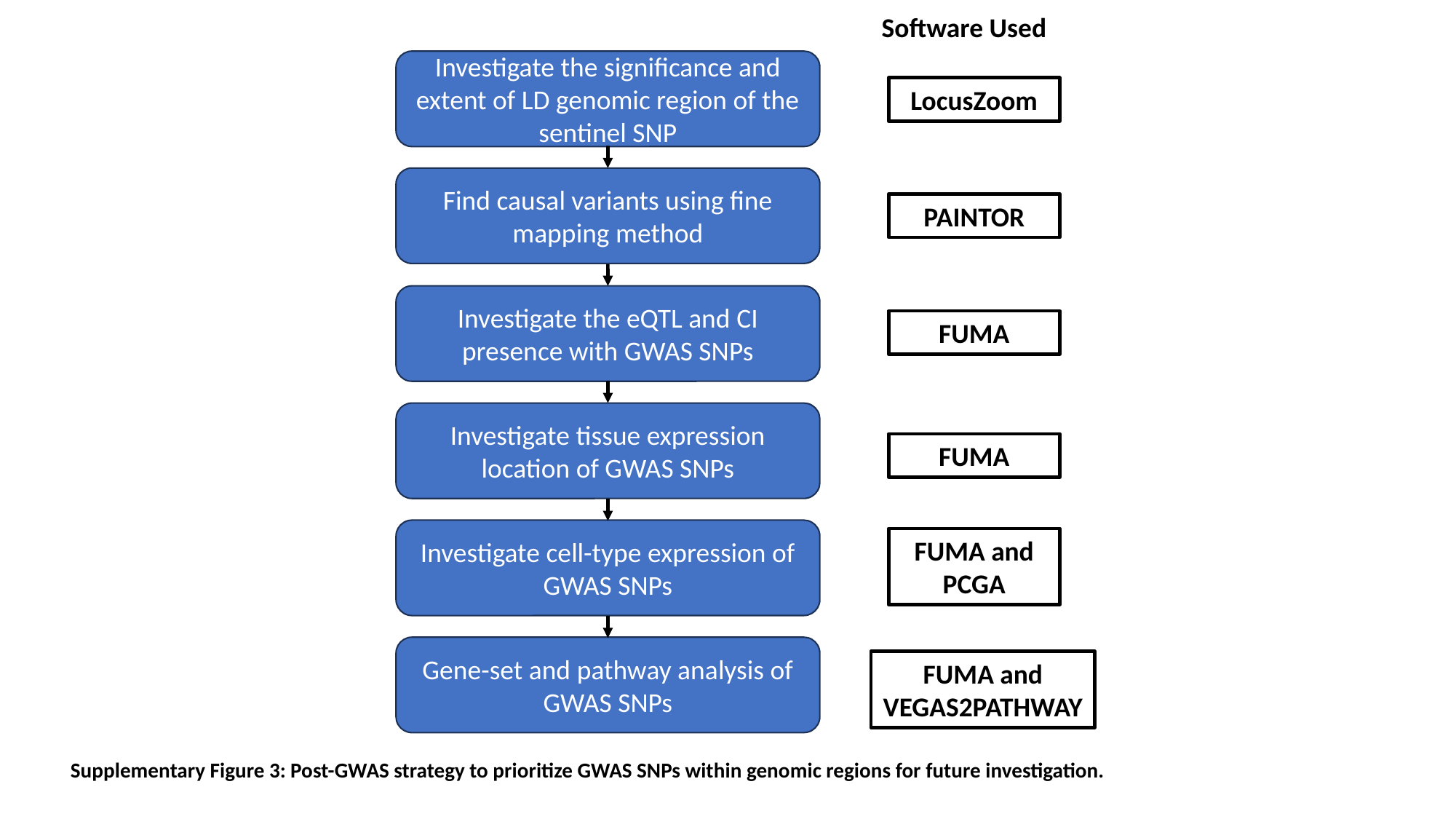

Software Used
Investigate the significance and extent of LD genomic region of the sentinel SNP
LocusZoom
Find causal variants using fine mapping method
PAINTOR
Investigate the eQTL and CI presence with GWAS SNPs
FUMA
Investigate tissue expression location of GWAS SNPs
FUMA
Investigate cell-type expression of GWAS SNPs
FUMA and PCGA
Gene-set and pathway analysis of GWAS SNPs
FUMA and VEGAS2PATHWAY
Supplementary Figure 3: Post-GWAS strategy to prioritize GWAS SNPs within genomic regions for future investigation.

## Slide 4
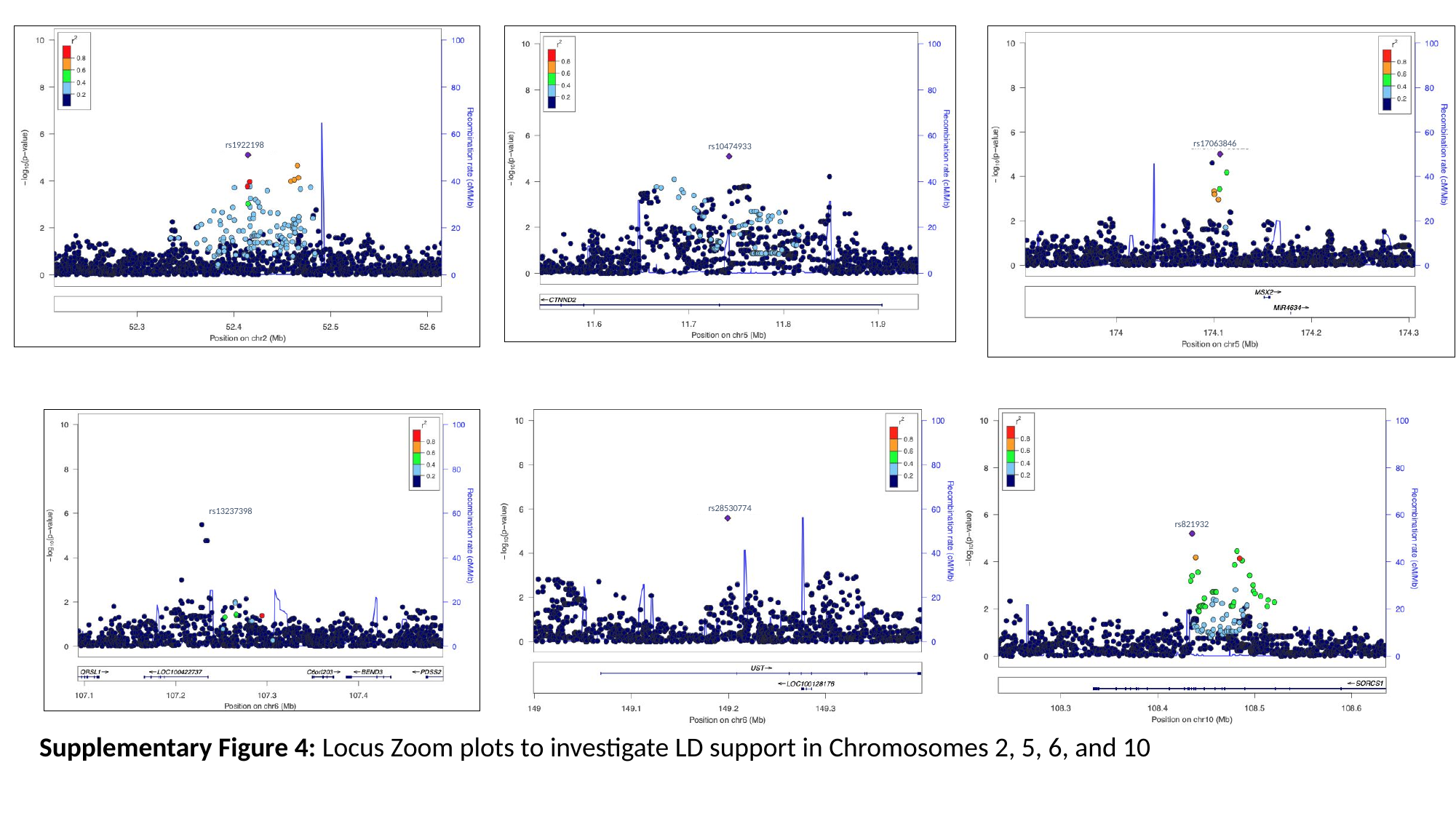

rs1922198
rs10474933
rs17063846
rs28530774
rs821932
rs13237398
Supplementary Figure 4: Locus Zoom plots to investigate LD support in Chromosomes 2, 5, 6, and 10

## Slide 5
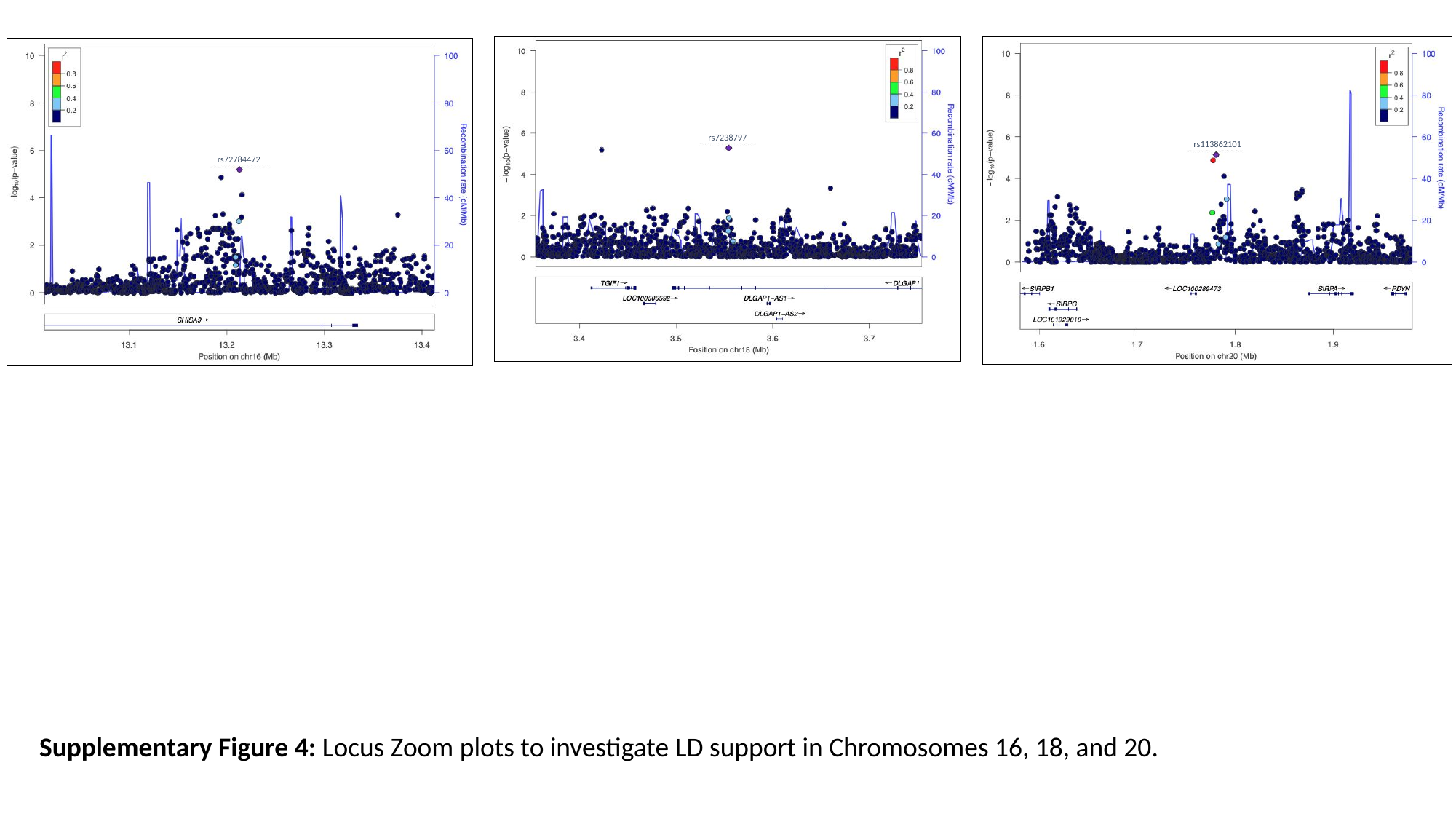

rs7238797
rs72784472
rs113862101
Supplementary Figure 4: Locus Zoom plots to investigate LD support in Chromosomes 16, 18, and 20.

## Slide 6
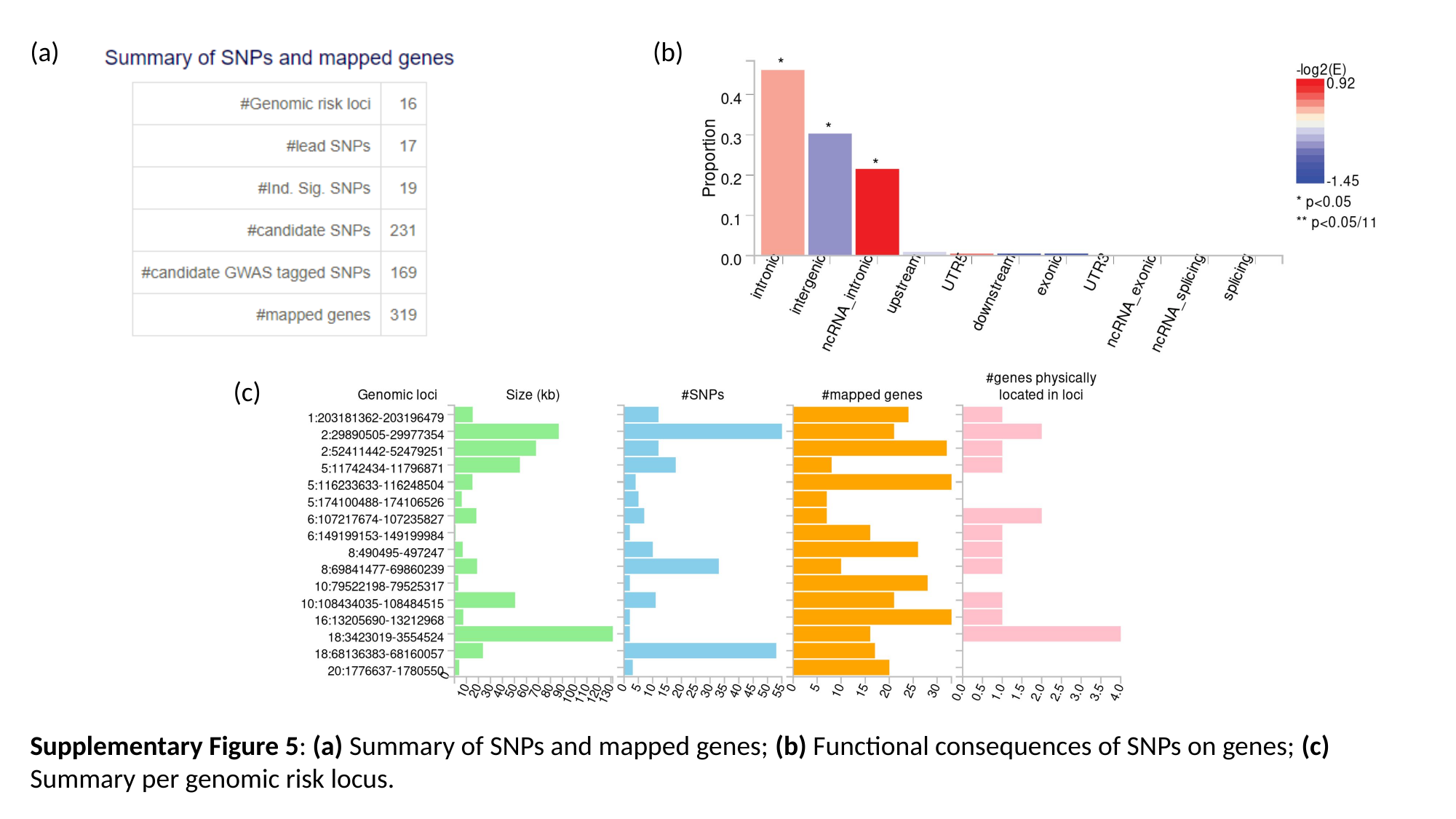

(b)
(a)
(c)
Supplementary Figure 5: (a) Summary of SNPs and mapped genes; (b) Functional consequences of SNPs on genes; (c) Summary per genomic risk locus.

## Slide 7
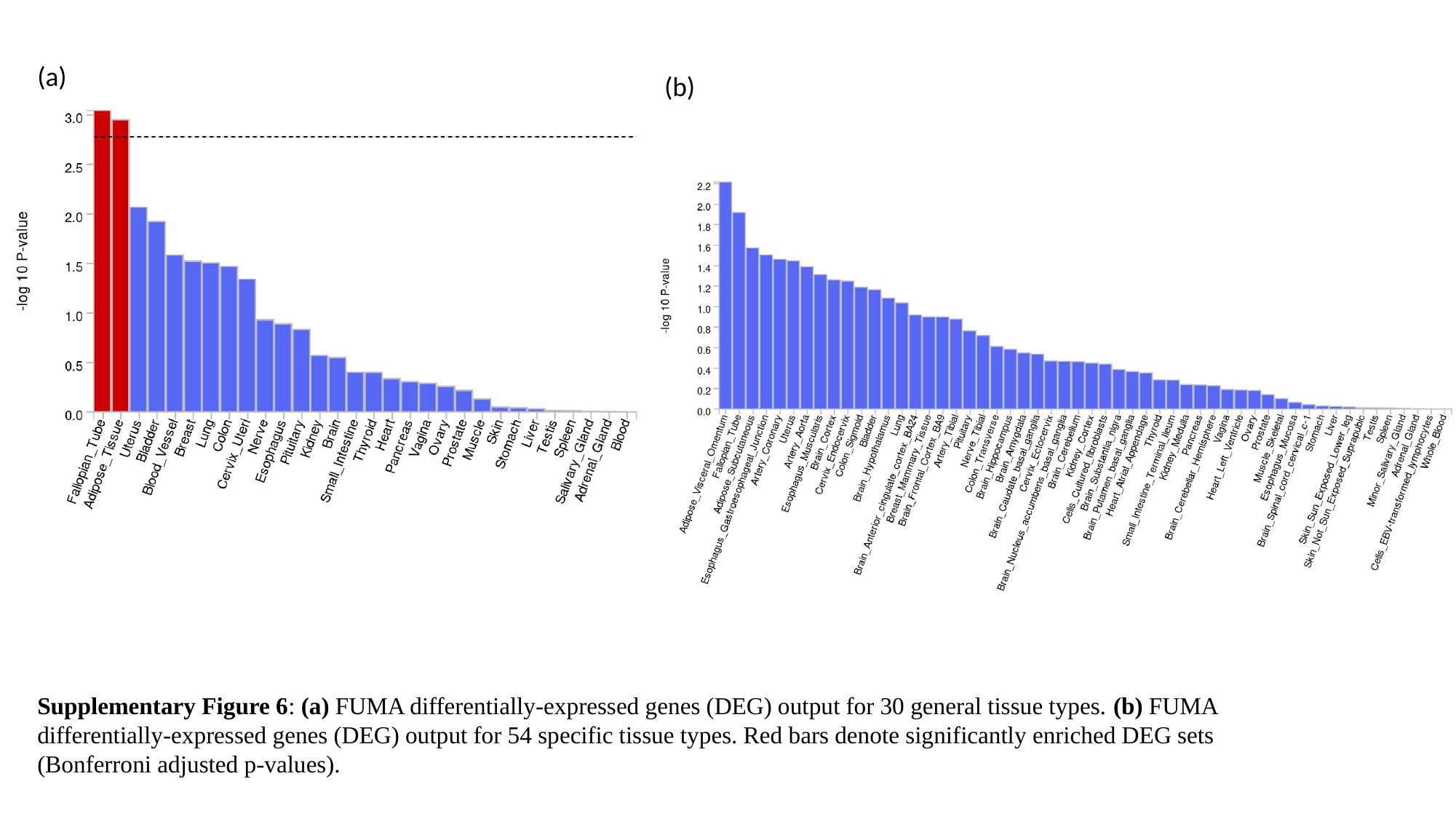

(a)
(b)
Supplementary Figure 6: (a) FUMA differentially-expressed genes (DEG) output for 30 general tissue types. (b) FUMA differentially-expressed genes (DEG) output for 54 specific tissue types. Red bars denote significantly enriched DEG sets (Bonferroni adjusted p-values).

## Slide 8
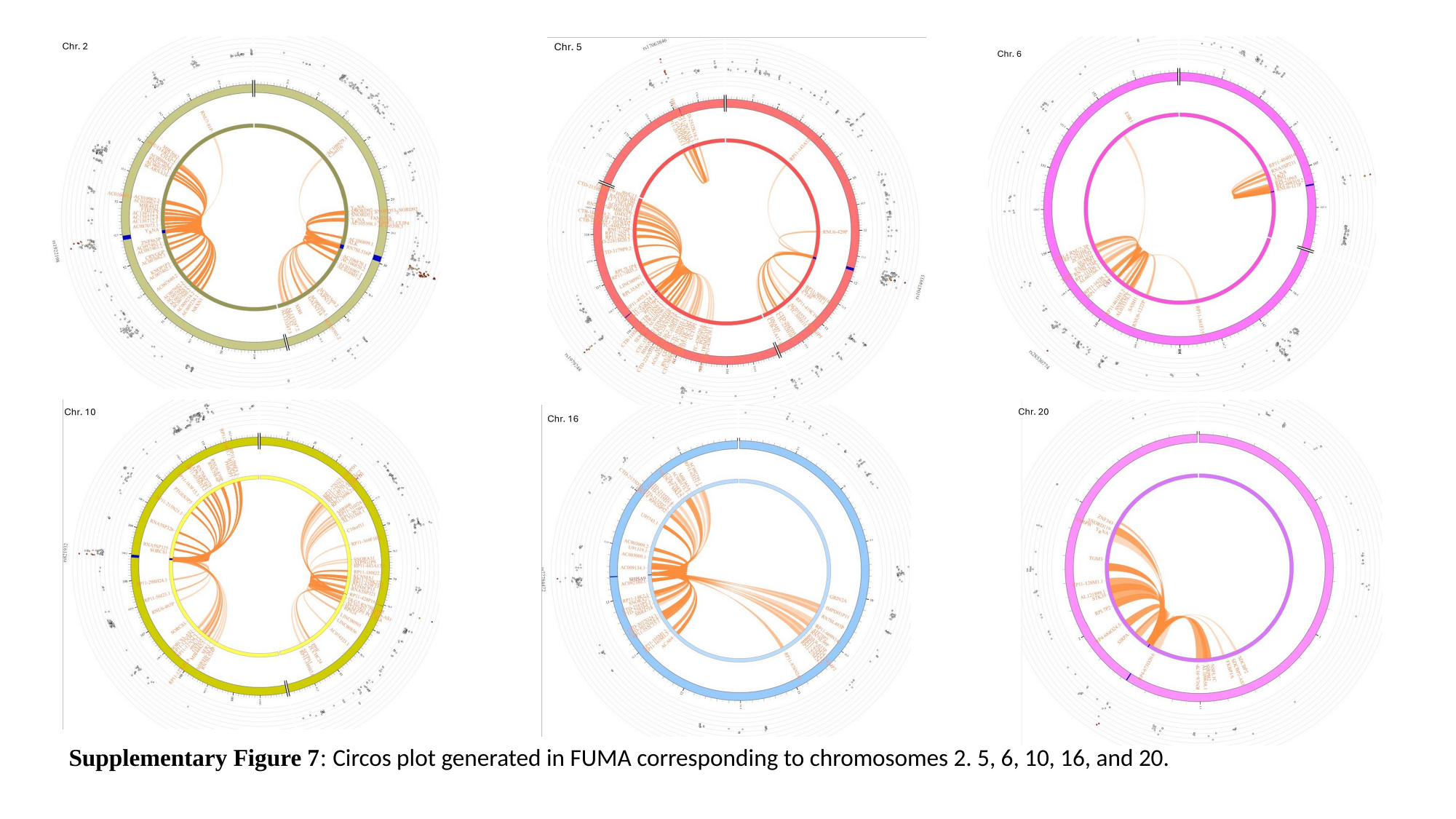

Supplementary Figure 7: Circos plot generated in FUMA corresponding to chromosomes 2. 5, 6, 10, 16, and 20.

## Slide 9
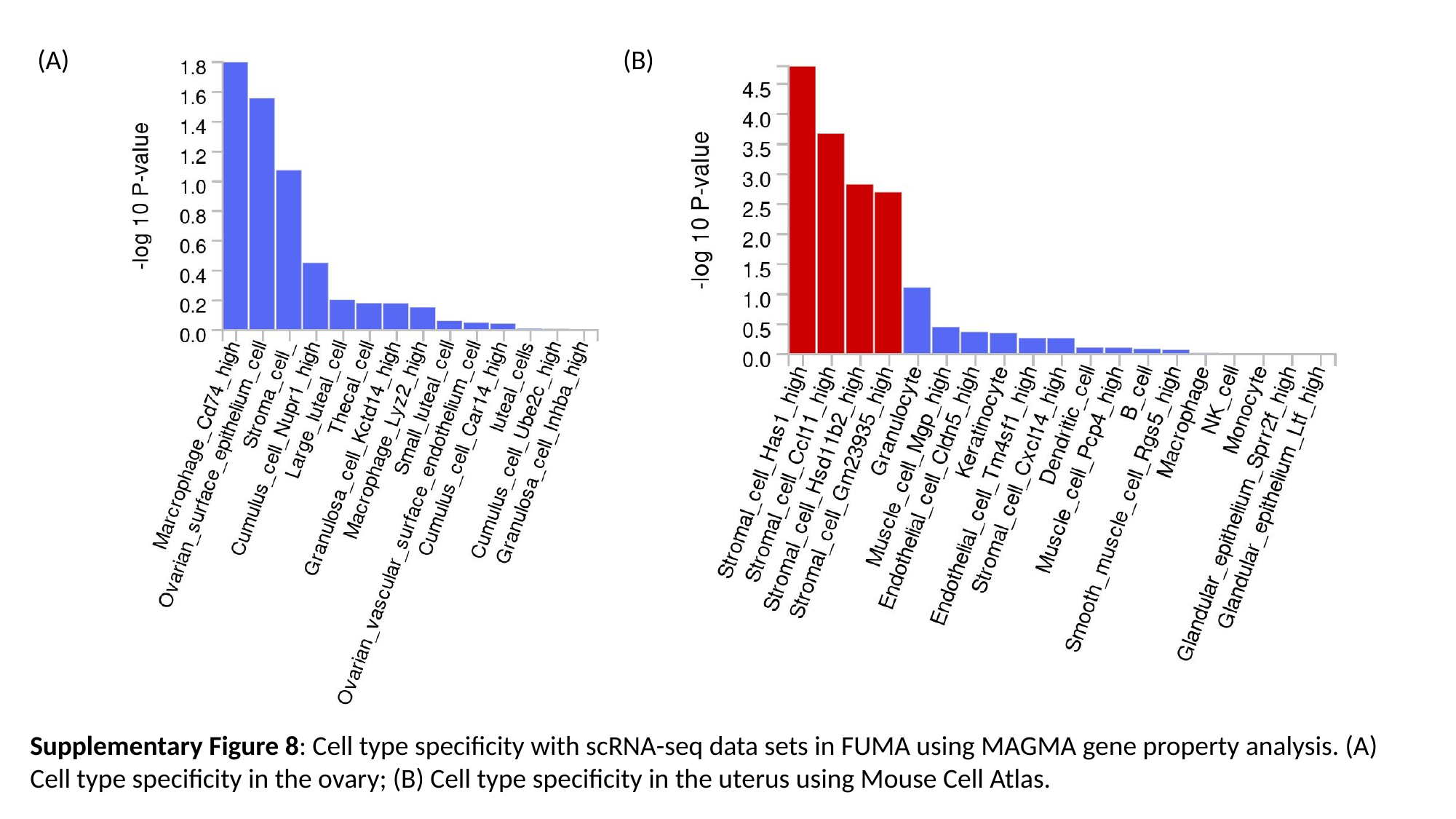

(A)
(B)
Supplementary Figure 8: Cell type specificity with scRNA-seq data sets in FUMA using MAGMA gene property analysis. (A) Cell type specificity in the ovary; (B) Cell type specificity in the uterus using Mouse Cell Atlas.

## Slide 10
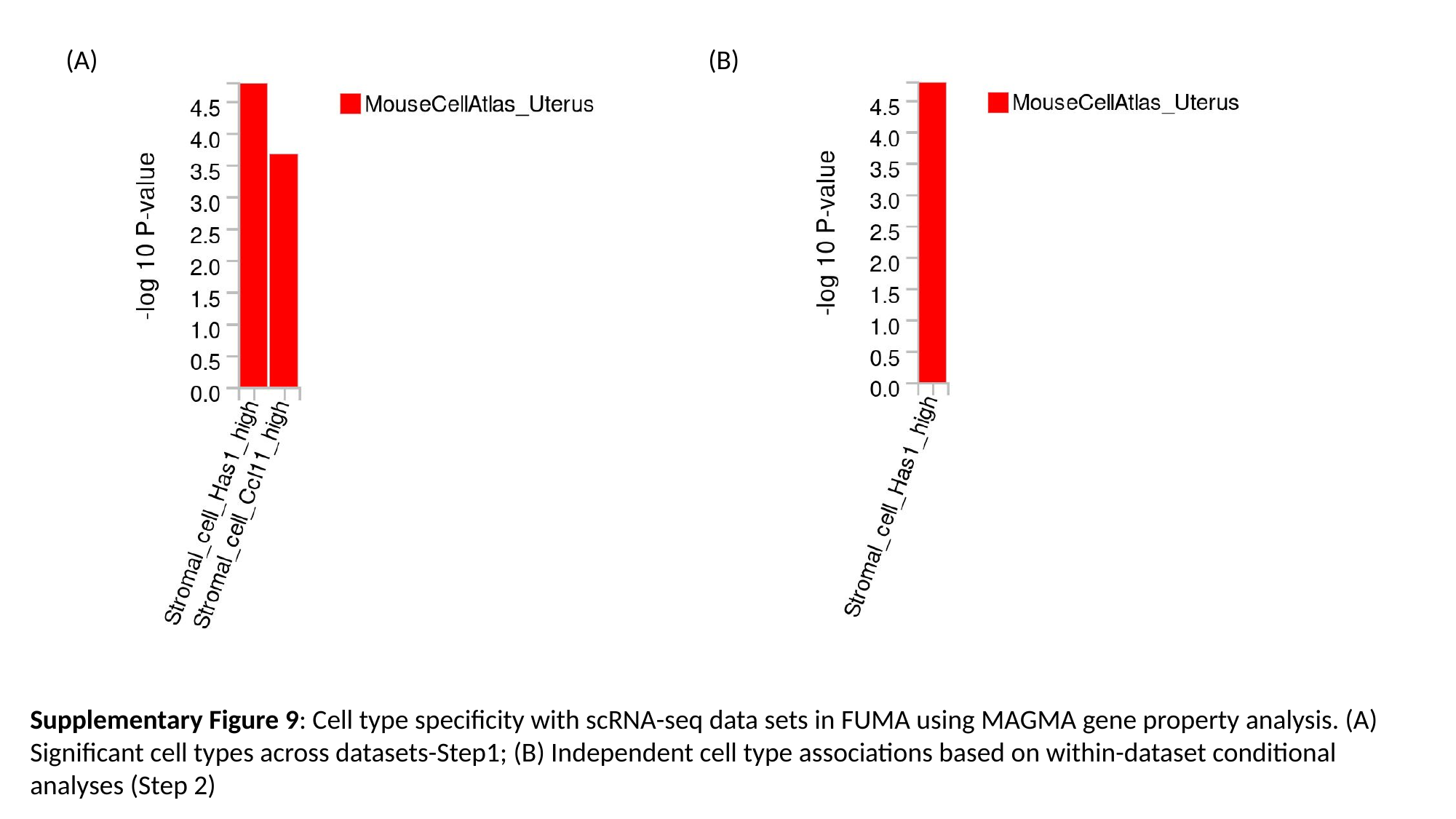

(A)
(B)
Supplementary Figure 9: Cell type specificity with scRNA-seq data sets in FUMA using MAGMA gene property analysis. (A) Significant cell types across datasets-Step1; (B) Independent cell type associations based on within-dataset conditional analyses (Step 2)
